# Mucoadhesive and Rheological Studies on the Co-Hydrogel Systems of Poly(Ethylene Glycol) Copolymers with Fluoroalkyl and Poly(Acrylic Acid)

**DOI:** 10.3390/polym13121956

**Published:** 2021-06-12

**Authors:** Yang Sun, Adiel F. Perez, Ivy L. Cardoza, Nina Baluyot-Reyes, Yong Ba

**Affiliations:** Department of Chemistry and Biochemistry, California State University, Los Angeles, CA 90032, USA; youngsoon1987@gmail.com (Y.S.); apere330@calstatela.edu (A.F.P.); icardoz@calstatela.edu (I.L.C.); nbaluyo@calstatela.edu (N.B.-R.)

**Keywords:** poly(ethylene glycol), fluoroalkyl, poly(acrylic acid), hydrogels, mucoadhesion, tensile strength, rheology, pH sensitivity

## Abstract

A self-assembled co-hydrogel system with sol-gel two-phase coexistence and mucoadhesive properties was developed based on the combined properties of fluoroalkyl double-ended poly(ethylene glycol) (R_f_-PEG-R_f_) and poly(acrylic acid) (PAA), respectively. We have synthesized an R_f_-PEG-g-PAA (where g denotes grafted) copolymer and integrated it into the R_f_-PEG-R_f_ physically cross-linked micellar network to form a co-hydrogel system. Tensile strengths between the co-hydrogel surfaces and two different sets of mucosal surfaces were acquired. One mucosal surface was made of porcine stomach mucin Type II, while the other one is a pig small intestine. The experimental results show that the largest maximum detachment stresses (MDSs) were obtained when the R_f_-PEG-g-PAA’s weight percent in the dehydrated polymer mixture is ~15%. Tensile experiments also found that MDSs are greater in acidic conditions (pH = 4–5) (123.3 g/cm^2^ for the artificial mucus, and 43.0 g/cm^2^ for pig small intestine) and basic conditions (pH = 10.6) (126.9 g/cm^2^, and 44.6 g.cm^2^, respectively) than in neutral pH (45.4 g/cm^2^, and 30.7 g.cm^2^, respectively). Results of the rheological analyses using shear strain amplitude sweep and frequency sweep reveal that the R_f_-PEG-g-PAA was physically integrated into the R_f_-PEG-R_f_ micellar network, and the co-hydrogels remain physically cross-linked in three-dimensional micellar networks with long-term physical dispersion stability. Therefore, the co-hydrogel system is promising for drug delivery applications on mucosal surfaces.

## 1. Introduction

Mucoadhesive biocompatible materials are desirable for localized drug-delivery applications on mucosal surfaces for maintaining sustained bioavailability of drugs [1,2,3]. Poly(ethylene glycol) (PEG)-based hydrogel materials with mucoadhesive properties are especially applicable due to PEG’s biocompatibility [4,5,6,7,8]. It is known that acrylic-based polymers have high adhesive bond strengths with mucus [9,10,11]. Therefore, hydrogels containing poly(acrylic acid) (PAA) are among the most recognized mucoadhesive systems and have been widely used in topical and oral drug deliveries [12,13]. In addition, PAA based polymers exhibit pH response [14,15]. Mucus is a viscous, adherent secretion which lines all body cavities exposed to the external environment. The main component of mucus are mucins, which are a class of high molecular weight (0.5 × 10^6^ to 20 × 10^6^) glycoproteins [16,17]. Mucins are responsible for the viscoelastic gel-like property of mucus, and for the adhesive interaction with the PAA component. 

Hydrogels are three-dimensional (3D) networks formed by cross-linked hydrophilic polymers [8,15,18,19]. The crosslinking agents, either physical or chemical, are essential to stabilize the hydrogels against solubilization when swelling in aqueous solutions [20,21,22]. It is challenging to maintain a physically cross-linked hydrogels with long-term stability in water compared to those which are chemically cross-linked. Therefore, physically cross-linked hydrogels with self-assembled gel phase in equilibrium with the sol phase are important for sustained drug release. Physically cross-linked hydrogels formed by self-assembly of fluoroalkyl double-ended poly(ethylene glycol) (previously abbreviated as double-ended R_f_-PEG, here referred to as R_f_-PEG-R_f_) have been reported to have such a property [23,24,25]. It was shown that the gel’s concentration and viscoelastic property vary with the PEG midblock length and the R_f_-end group’s molecular weight. An interesting feature of this system is that the gel phase can maintain equilibrium composition in water with surface erosion. In this study, a co-hydrogel system with the properties of sol-gel two-phase coexistence and mucoadhesion has been developed based on the combined properties of R_f_-PEG-R_f_ and acrylic acid-based polymers, respectively. We have synthesized an R_f_-PEG-g-PAA (where g denotes grafted) copolymer and integrated it into the R_f_-PEG-R_f_ hydrogel network through self-assembly. Our experimental data show that the R_f_-PEG-R_f_/R_f_-PEG-g-PAA co-hydrogel system possesses both gel moduli and mucoadhesive properties. Therefore, the co-hydrogel system is promising to be developed as a drug-delivery platform for sustained drug deliveries on mucosal surfaces. 

Formation of the R_f_-PEG-R_f_ gel network is attributed to the R_f_-groups that are both hydrophobic and lipophobic. The R_f_-groups at both ends of the PEG chains fold together to form the hydrophobic cores of the micelles, meanwhile pairs of R_f_-groups of other R_f_-PEG-R_f_ chains associate with the R_f_-cores to crosslink the neighboring micelles to form a micellar network in an aqueous environment. Results of a SANS (small angle neutron scattering) experiment shows that R_f_-PEG-R_f_ formed a soft ordered lattice in water [24]. In this study, we hypothesized that the R_f_-group of the R_f_-PEG-g-PAA can self-assemble with the R_f_-cores of the R_f_-PEG-R_f_ micelles. Therefore, the gel-surface extruding PAA blocks of the co-hydrogel can interact with mucin, resulting in mucoadhesive properties. Assembly of the co-hydrogel system and its interaction with a mucus membrane is schematically shown in the graphical abstract of this paper. As will be demonstrated in this paper, the R_f_-PEG-R_f_/R_f_-PEG-g-PAA can self-assemble to form a co-hydrogel. It is interesting that the co-hydrogel materials are also soluble in many organic solvents such as methanol and dichloromethane. Therefore, the co-hydrogel system allows for flexible formulation of drug-loaded, or nanoscale drug carrier-loaded, compositions for drug-delivery applications. 

Tensile strength testing has been used to study the interactions between the mucoadhesive materials and mucosal surfaces [14,26,27,28]. In this study, we have also applied this method to study the adhesive interactions between R_f_-PEG-R_f_/R_f_-PEG-g-PAA co-hydrogels with the surfaces of an artificial mucus made of porcine stomach mucin Type II and a pig small intestine, respectively. Rheological methods are informative to understand the physical and chemical cross-linkages and mechanical properties of hydrogels [29,30,31,32,33,34,35]. Therefore, we have used rheological methods to study the co-hydrogel system with presets of shear strain and frequency deformation. The results revealed the structural integrities of the co-hydrogels with various amounts of R_f_-PEG-g-PAA integrated in the R_f_-PEG-R_f_ hydrogel networks in different pH conditions. 

## 2. Materials and Methods

### 2.1. Materials

Polyethylene glycol (abbreviated as PEG, molecular weight (MW) = 6.0 × 10^3^, CAS Number 25322-68-3), poly(acrylic acid) (abbreviated as PAA, MW = 1.8 × 10^3^, CAS Number 9003-01-4), 1h,1h,2h,2h-perfluorooctanol (abbreviated as R_f_-OH, CAS Number 647-42-7), isophorone diisocyanate (abbreviated as IPDI, CAS Number 4098-71-9), dibutyltin dilaurate (abbreviated as DBTDL, CAS Number 77-58-7), Tetrahydrofuran (abbreviated as THF), anhydrous (CAS Number 109-99-9), 3-Indoleacrylic acid (abbreviated as IAA, CAS Number 1204-06-4)**,** methanol-d4 (CAS Number 811-98-3) and porcine stomach mucin Type II (CAS Number 84082-64-4) were all purchased from Sigma-Aldrich, St. Louis, MI, USA. Reagent grade solvents including ethylene glycol dimethyl ether anhydrous (commonly called glyme, CAS Number 110-71-4), diethyl ether anhydrous (CAS Number 60-29-7), methanol (CAS Number 67-56-1), hexane (HPLC grade, CAS Number 110-54-3), 1-ethyl-3-(3-dimethylaminopropyl) carbodiimide hydrochloride (abbreviated as EDC-HCl, CAS Number 25952-53-8), *n*-hydroxysuccinimide (abbreviated as NHS, CAS Number 6066-82-6), *n*,*n*-diisopropylethylamine (abbreviated as DIEA, CAS Number7087-68-5), and anhydrous dimethylformamide (abbreviated as DMF, CAS Number 68-12-2) were all obtained from Fisher Scientific, Hampton, NH, USA. Sodium hydroxide (CAS Number 1310-73-2), sodium phosphate dibasic anhydrous (CAS Number 7558-79-4), sodium phosphate monobasic anhydrous (CAS Number 7558-80-7), Slide-A-Lyzer (7.0 K MWCO) dialysis cassette (Catalog No. PI66710) were all obtained from Fisher Scientific, USA. The PEG and R_f_-OH were further dried under a high vacuum, and IPDI was further purified through vacuum distillation before use. We prepared 5 wt% artificial mucus through dissolving 0.5 g mucin in 9.5 mL buffer in a 50 mL Falcon tube followed by a vortex for 15 mins. Afterwards, the artificial mucus was annealed for 1 h before use. A pig small intestine was provided by BioreclamationIVT, Westbury, NY, USA. The small intestine was rinsed with water before shipped to our lab in frozen state.

### 2.2. Synthesis of Double Ended R_f_-PEG (Fluoroalkyl Double-Ended Poly(Ethylene Glycol) (R_f_-PEG-R_f_))

PEG with MW = 6.0 × 10^3^ and the R_f_-OH were used to synthesize R_f_-PEG-R_f_. Procedure for the synthesis has been described in other’s and our previous publications [23,24,36,37,38,39]. Briefly, the interconnection of the R_f_-OH alcohol group with both PEG’s OH ends were attained through the IPDI linker. The R_f_-IPMU (isophorone monourethane) intermediate was first obtained through the reaction of the R_f_-OH with an excess molar amount of IPDI. To produce the R_f_-PEG-R_f_ polymer, freshly vacuum-dried PEG and an excess molar amount of R_f_-IPMU were dissolved in glyme, and a few drops of DBTDL were added to catalyze the reaction followed by purification. A yield of 76% was obtained. The route of synthesis was given in Scheme S1 of the supplementary file.

### 2.3. Synthesis of R_f_-PEG-g-PAA (Poly(Acrylic Acid))

The R_f_-PEG-OH was obtained through the same procedure as that of R_f_-PEG-R_f,_ but with the molar ratio of R_f_-IPMU to PEG adjusted between 1 and 2. The yield of the R_f_-PEG-OH product was ~78%. The R_f_-PEG-OH was then grafted to the PAA. Briefly, 0.1 mmol (0.180 g) of PAA, 0.15 mmol (0.029 g) of EDC-HCl, and 0.15 mmol (0.017 g) of NHS were dissolved in 6 mL anhydrous DMF in a 25 mL round bottom flask. The solution was then stirred at room temperature overnight to form the PAA-NHS ester. Then, 0.1 mmol (0.659 g) R_f_-PEG-OH ($\mathrm{average}\;\mathrm{molecular}\;\mathrm{weight},\;\overset{——}{\mathrm{MW}}=6586.41$), 0.25 mmol (0.0323 g) of DIEA, and another 6 mL anhydrous DMF were added into the PAA-NHS ester solution. The solution was stirred for four days at room temperature to obtain the grafted R_f_-PEG-g-PAA copolymer. Route of the synthesis is shown in Scheme S2 of the supplementary file. Dialysis was used to purify the product, and lyophilization was used to obtain the product, R_f_-PEG-g-PAA. The yield was found to be ~65%.

### 2.4. Preparation of the R_f_-PEG-R_f_ and R_f_-PEG-g-PAA Co-Hydrogels

A homogeneous solid mixture of R_f_-PEG-g-PAA and R_f_-PEG-R_f_ was made by dissolving both in methanol and then lyophilizing it overnight. The lyophilized solid powder was compressed to a tablet followed by being immersed in water or other buffer solutions. For example, a tablet of 320 mg of the dried 5 wt% R_f_-PEG-g-PAA and 95 wt% R_f_-PEG-R_f_ mixture was placed in 11.0 mL DI water (pH = 4–5 due to the PAA), 11.0 mL phosphate-buffered saline (PBS) buffer (pH = 7.2), or 11.0 mL Glycine/NaOH buffer (pH = 10.6). Seven days were used to homogenize the co-hydrogel at 37 °C. The gel phase was separated from the sol phase using an automatic pipette. Table 1 summarizes the compositions of R_f_-PEG-g-PAA, and R_f_-PEG-R_f_ for making the co-hydrogels. PAA was also used to mix with the R_f_-PEG-R_f_ as a control experiment to prove the association of the R_f_-group in the R_f_-PEG-g-PAA with the R_f_-cores of the R_f_-PEG-R_f_ micelles in the tensile strength test. Deionized (DI) water, PBS, glycine/NaOH buffer solution were used to make the co-hydrogels. 

### 2.5. Molecular Weight and Structural Characterizations Using Matrix-Assisted Laser Desorption/Ionization-Time of Flight (MALDI-TOF) Mass Spectrometry (MS) and Nuclear Magnetic Resonance (NMR) Instruments

Matrix-assisted laser desorption/ionization-time of flight (MALDI-TOF) is a soft ionization technique that enables the measurement of molecular weight distribution of polymers [40]. Both number average molecular weight (M_n_) and mass average molecular weight (M_w_), thus the polydispersity indexes (PDI = M_w_/M_n_) can be determined from the mass distribution of the spectra [41]. A MALDI-TOF mass spectrometry (MS) instrument, Voyager-DE STR, (Applied Biosystems, Waltham, MA, UAS), was used to characterize PEG, R_f_-PEG-R_f_, R_f_-PEG-OH and R_f_-PEG-g-PAA. To undertake the MS experiment, 0.25 M M indole-3-acetic acid (IAA) in THF solvent was prepared as the matrix solution. 10 g/L concentrations of the polymer solutions were made in DI water for PEG, and in methanol for R_f_-PEG-R_f_, R_f_-PEG-OH, and R_f_-PEG-g-PAA. Volume ratio of 1:1 of the sample solution to the matrix solution was used. One μL of the mixed solution was loaded into a well of the MOLDI-TOF plate. ^1^H NMR (nuclear magnetic resonance) spectra were acquired using a Bruker BioSpin Avance^I^ (Billerica, MA, USA) 400 MHz NMR instrument to characterize the structure of R_f_-PEG-g-PAA. Methanol-d_4_ (CD_3_OD) was used as the NMR solvent.

### 2.6. Mucoadhesion Study by Tensile Strength Method

Investigation of the mucoadhesive bond strength between the surfaces of the co-hydrogels and the surfaces of the artificial mucus and the pig small intestine was carried out using the tensiometric technique [14,26,27,28]. In a typical tensile experiment, the force necessary for detachment of the two surfaces is recorded as a function of elongation observed at the polymer–mucus interface. The stress is equal to the force divided by the total initial area, and the work of adhesion is calculated as the area under the force-elongation curves. Here, we compared the maximum detachment stresses (MDSs) (maximum forces required to separate the two surfaces divided by the areas) among the different compositions of the co-hydrogels with the mucosal surfaces. A TA.XTplus texture analyzer (Stable Micro Systems, Godalming, Surrey, UK) was used for doing the experiments and the Exponent software from Stable Micro Systems was used for analyzing the data. For the mucoadhesion study with the mucus made of the porcine stomach mucin Type II [42], a super strong waterproof black adhesive double-sided foam mounting tape was used. A piece of 25 mm × 25 mm of the double-sided tape was placed on the center of the platform (bottom), and then covered with a 25 mm × 25 mm filter paper. Also, a piece of 10 mm × 10 mm double-sided tape was adhered to the center of the round probe (top) to which 10 mm × 10 mm filter paper was mounted (see Figure S1 in the Supplementary Information.) A 30 µL co-hydrogel sample was placed onto the 10 mm × 10 mm filter paper, and a 185 µL mucus sample was applied onto the 25 mm × 25 mm filter paper. The samples were allowed to homogenize for 5 min in the filter papers before running each test. For the mucoadhesion study with the pig small intestine, a 25 mm × 25 mm pig small intestine tissue with internal surface (the surface covered with mucosal layer) up was placed on the surface of the texture analyzer’s platform [14,43]. A zinc plated cut washer with 3/8 inches in inner-diameter was placed on top of the small intestine sample to secure the pig small intestine tissue sticking on the platform surface (see Figure S2). A 0.5 inch in diameter double sided tape was adhered to the center of the TA-10 probe. The bottom side of the double sided tape was covered with a 0.5 inch in diameter filter paper, which was then soaked with the co-hydrogel sample (see Figure S2). When running the tensile strength experiment, once a trigger force of 150 g was detected on the surface of the sample, the probe proceeded to compress the sample until a 250 g force was reached. The force is then held for 60 s to let the co-hydrogels have enough contact time with the mucus surface. The probe then withdrew to a maximum distance of 11 mm above the sample. The test of each sample was repeated at least 4 times to ensure repeatability.

### 2.7. Rheological Measurements

Rheological measurements were carried out using a modular compact Rheometer (MCR 302, Anton Paar GmbH, Graz, Austria). An electrically heated plate temperature system was set at 37 °C to mimic human physiological temperature. To prevent the wall-spill effect, a 25 mm diameter sandblasted parallel plate (PP25/S, Anton Paar) geometry was used. The hydrogel sample was placed between the two plates, and the width of the gap between the parallel plates was set to 0.5 mm. A solvent trap device was used to prevent the evaporation of water in the hydrogels. Two minutes were used to homogenize the sample temperature before starting the tests. Shear strain amplitude sweeps and frequency sweeps were carried out to characterize the viscoelastic properties of the co-hydrogels. Shear stress versus shear strain curves and moduli versus shear strain curves were acquired by shear strain amplitude sweeps using a low frequency of 10 s^−1^. The frequency sweeps were carried out to measure G’ and G” moduli as functions of angular frequency ranging from 100 rad/s to 0.1 rad/s. A shear strain of 1% in the linear viscoelastic (LVE) range was used during the frequency sweep. The measurements were carried out using logarithmic steps and the corresponding logarithmic scale was also plotted to illustrate the values in both small and large scales. 

## 3. Results and Discussion

### 3.1. Chemical Analysis of the Product

MALDI-TOF MS spectrum of the PEG is shown in Figure S3, and that of the R_f_-PEG-R_f_ is shown in Figure S4. The average molecular weight of the PEG was determined to be 6.2 × 10^3^ (PDI = 1.005), and that of the R_f_-PEG-R_f_ to be ~7.4 × 10^3^ (PDI = 1.006), showing the successful synthesis of the R_f_-PEG-R_f_. The MALDI TOF MS spectrum of the R_f_-PEG-OH is shown in Figure S5. The molecular weight of this intermediate is ~6.8 × 10^3^ (PDI = 1.007) showing the produced R_f_-PEG-OH. The MALDI TOF MS spectrum of the R_f_-PEG-g-PAA is shown in Figure S6, which has an average molecular weight of 8.0 × 10^3^ (PDI = 1.011) showing the successful synthesis of the R_f_-PEG-g-PAA product. Figure S7 shows the ^1^H NMR spectrum of the R_f_-PEG-g-PAA. The major peak at 3.5407 ppm shows the PEG block, –[CH_2_CH_2_O]–. The PAA ^1^H signals are quite broadly distributed from 1.0 ppm to 2.5 ppm which can only be seen after the relative intensity of the spectrum is scrolled up. Figure S8 shows the comparison of the spectra of R_f_-PEG-g-PAA (bottom, red) and PAA (top, blue) in the PAA region, which indicates the PAA block in the R_f_-PEG-g-PAA copolymer.

### 3.2. Mucoadhesion by Tensile Tests

Figure S9 shows the photos of the sol-gel two-phase coexistences of the 5% R_f_-PEG-g-PAA/95% R_f_-PEG-R_f_ (composition #2), and the 10% R_f_-PEG-g-PAA/90% R_f_-PEG-R_f_ (composition #4), respectively, prepared in the following buffers or water at 37 °C after 11 days: (A) and (B) in PBS buffer (pH = 7.2); (C) and (D) in DI water (pH = 4–5); and (E) and (F) in the glycine/sodium hydroxide buffer (pH = 10.6). The volumes of the gel phases did not expand further after several days. These results demonstrate that the sol-gel two-phase coexistences in these buffers were retained after long incubation periods. The gel phases were used to conduct the mucoadhesion experiments.

Figure S10 shows the force (g) vs. time (s) curves for the 5.0% mucin Type II artificial mucus sample interacting with the R_f_-PEG-g-PAA/R_f_-PEG-R_f_ co-hydrogels (compositions #2 to #6 in Table 1) and the control samples (water, composition #1 and compositions #7 to #10) prepared in water. The maximum detachment stresses (MDSs) (g/cm^2^) required to separate the two surfaces can be seen on the tops of the peaks divided by the surface area. To make the MDSs more visible, Figure S11 shows the relative MDSs with respect to the MDS of the water sample, i.e., the relative MDS = MDS (sample)-MDS (water). The MDS of the water sample with the artificial mucus sample is the lowest compared to all the other samples. The relative MDS increased with the increase of the R_f_-PEG-g-PAA component until the sample of 15.0% R_f_-PEG-g-PAA/85.0% R_f_-PEG-R_f_ (composition #6). After surpassing 15.0% Rf-PEG-g-PAA, the relative MDS decreased as shown by the sample of 20.0% R_f_-PEG-g-PAA/80.0% R_f_-PEG-R_f_ (composition #7). The above result proves that with low R_f_-PEG-g-PAA percentage, the R_f_-PEG-g-PAA could be held strongly in the co-hydrogel phase. The gel surface-bound PEG-g-PAA blocks protruded into the water phase to interact with mucin in the mucus phase. To see if the R_f_-group in R_f_-PEG-g-PAA truly played a role in holding the -PAA block on the surface of the co-hydrogel, experiments were performed using 5.0% PAA/95.0% R_f_-PEG-R_f_ (composition #8) and the 10.0% PAA/90.0% R_f_-PEG-R_f_ (composition #9). As expected, the relative MDSs of these PAA/R_f_-PEG-R_f_ samples dramatically decreased compared to all the R_f_-PEG-g-PAA/R_f_-PEG-R_f_ samples. This result demonstrates that the R_f_-groups in R_f_-PEG-g-PAA physically associated with the R_f_-cores of the R_f_-PEG-R_f_ micelles. To demonstrate the necessity of R_f_-PEG-R_f_, experiments were also performed with the 5.0% R_f_-PEG-g-PAA (composition #10) and the 10.0% R_f_-PEG-g-PAA (composition #11) samples without R_f_-PEG-R_f_. Their relative MDSs are the smallest compared to all the other samples, proving that R_f_-PEG-g-PAA formed a co-hydrogel with R_f_-PEG-R_f_. In our opinion, it is difficult to give a regular range of MDS for mucoadhesion. In our opinion, besides the intrinsic mucoadhesive forces, the value of MDS also depends on the sample preparation in a study. Thus, comparison of the relative MDSs among a series of samples is more reliable than comparing data in publications of different authors.

Figure S12 shows the force vs. time curves for the 5.0% mucin Type II artificial mucus sample interacting with the R_f_-PEG-g-PAA/R_f_-PEG-R_f_ co-hydrogel and the control sample prepared in the PBS buffer. Figure S13 shows the corresponding relative MDSs with respect to the MDS of the PBS buffer sample. Figure S14 shows the force vs. time curves for the 5.0% mucin Type II artificial mucus sample interacting with the R_f_-PEG-g-PAA/R_f_-PEG-R_f_ co-hydrogel and the control sample prepared in the glycine/NaOH buffer. Figure S15 shows the corresponding relative MDSs with respect to the MDS of the glycine/NaOH buffer sample. The trends of the relative MDSs for the co-hydrogels in the PBS buffer and the glycine/NaOH buffer are the same as those of the co-hydrogel samples prepared in water. 

Figure S16 shows the force vs. time curves of the interactions of various R_f_-PEG-g-PAA/R_f_-PEG-R_f_ co-hydrogels and the control samples prepared in water with the pig small intestine. Figure S17 shows the corresponding bar graph presentation of the relative MDSs with respect to the water sample. The trends of the relative MDSs are the same as those of the co-hydrogel samples interacting with the mucus sample. The R_f_-PEG-R_f_ hydrogel alone, the R_f_-PEG-g-PAA hydrogel alone, and the R_f_-PEG-R_f_ hydrogel mixed with PAA also showed much lower MDSs compared to the R_f_-PEG-g-PAA/R_f_-PEG-R_f_ co-hydrogel samples. Figure S18 shows the force vs. time curves of the interactions of various R_f_-PEG-g-PAA/R_f_-PEG-R_f_ co-hydrogels and the control sample prepared in the PBS buffer with the pig small intestine. Figure S19 shows the corresponding bar graph presentation showing the corresponding relative MDSs. Figure S20 shows the force vs. time curves of the interactions of various R_f_-PEG-g-PAA/R_f_-PEG-R_f_ co-hydrogels and the control sample prepared in the glycine/NaOH buffer with the pig small intestine surface. Figure S21 shows the corresponding bar graph showing the relative MDSs. The trend of pH influence on MDS is the same as that seen in the experiments with the artificial mucus. 

**Figure 1:**
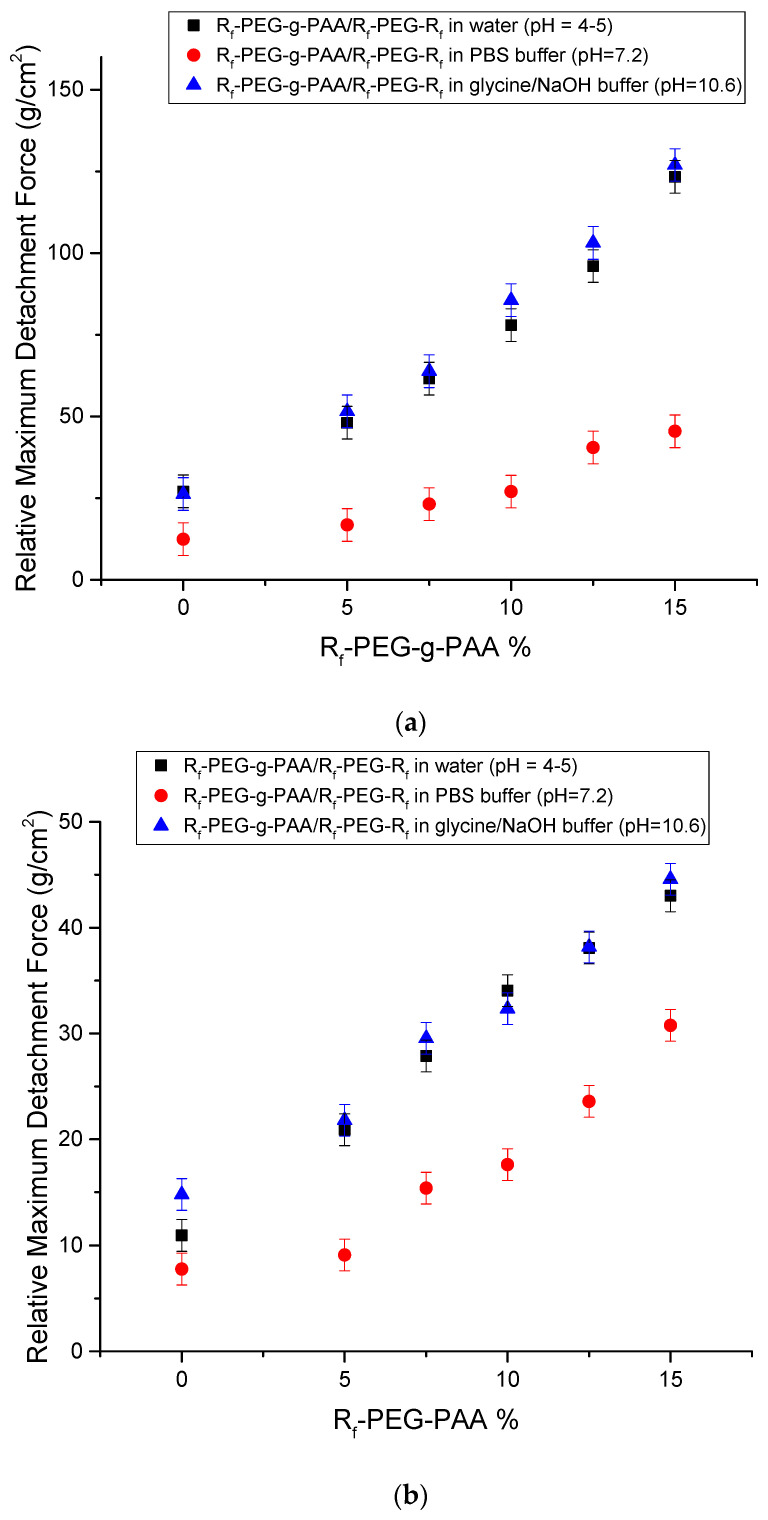
(**a**) Comparison of relative maximum detachment stresses (MDSs) of the interactions between the R_f_-PEG-g-PAA/R_f_-PEG-R_f_ samples and the mucus samples prepared in different buffers (shown in the inset). (**b**) Comparison of the relative MDSs of the interactions between the R_f_-PEG-g-PAA/R_f_-PEG-R_f_ samples prepared in different buffers (shown in the inset) and the pig small intestine surface.

Figure 1a shows the comparison of the relative MDSs of the interactions between the R_f_-PEG-g-PAA/R_f_-PEG-R_f_ co-hydrogel samples and the artificial mucus surface prepared in water and the buffers. Figure 1b shows the comparison of the relative MDSs of the interactions between the R_f_-PEG-g-PAA/R_f_-PEG-R_f_ hydrogel samples prepared in water and the buffers and the pig small intestine surface. The MDSs in both Figure 1a and b have the same trend. It shows that the adhesion forces of the co-hydrogels made in the glycine/NaOH buffer (pH = 10.6) and water (pH = 4–5) are larger than those made in the PBS buffer (pH = 7.2). Thus, the R_f_-PEG-g-PAA/R_f_-PEG-R_f_ co-hydrogels showed pH-sensitive mucoadhesion. The pH sensitivity indicates that hydrogen-bonding and electrostatic interactions between the PAA and the mucin played the major roles in the adhesions. 

### 3.3. Gel Structural Integrity by Rheological Analysis

#### 3.3.1. Shear Strain Amplitude Sweep

**Figure 2:**
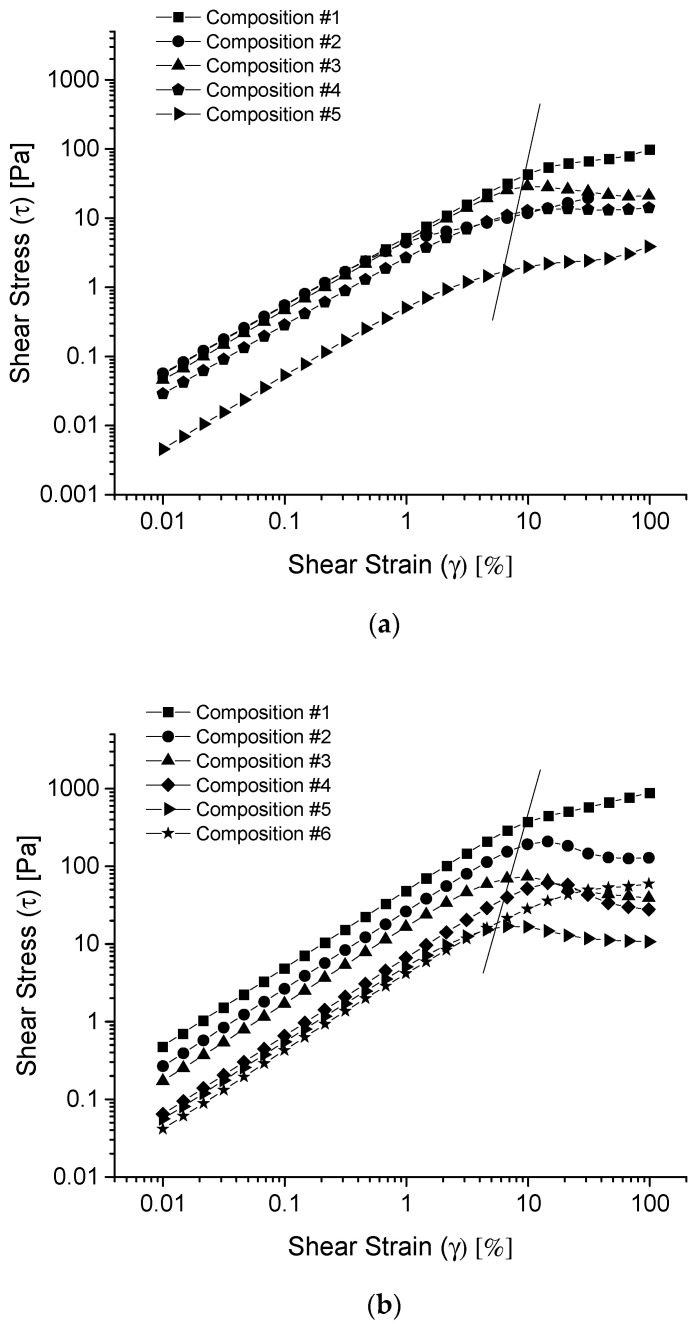

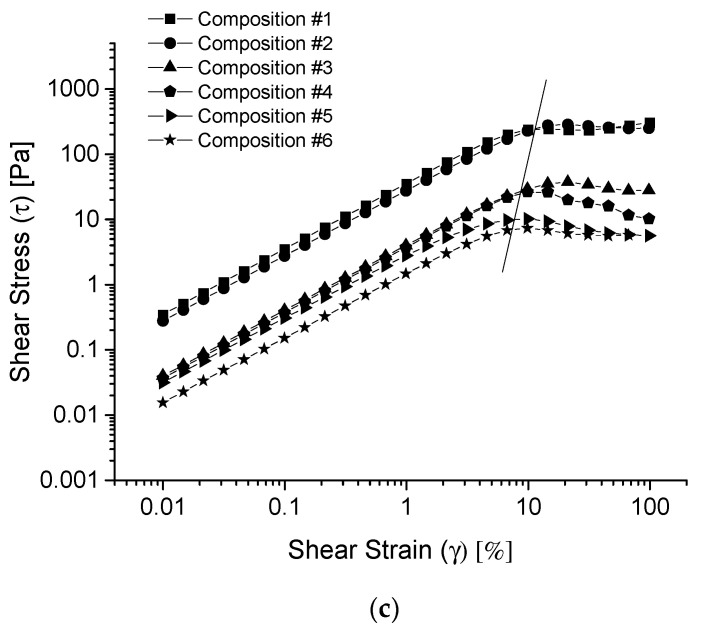
Shear stress (τ) versus shear strain (γ) curves (in logarithmic scales) for the given co-hydrogel systems prepared in water (**a**), phosphate-buffered saline (PBS) buffer (**b**), and glycine/NaOH buffer (**c**).

Amplitude sweep test provides information about the integrities/destructions of the internal structures of hydrogels at the preset shear strains or shear stresses. Figure 2 shows the shear stress versus shear strain curves (in logarithmic scales) for the given co-hydrogels prepared in water (a), PBS buffer (b) and glycine/NaOH buffer (c). Here, the shear strains (deformation of the co-hydrogel) are the preset in descending logarithmic steps, and the shear stresses were the responses to the shear strains. There are two regions in each of the curves. One is the initial linear-elastic (LE) region where the low shear stress increases linearly with the increase of the low shear strain. The other region is the flow region where the shear stress is off the LE curve. After the co-hydrogels could no longer sustain the added stress within the elastic limit, they yielded to the stress and, therefore, flowed. The cross-over point between the LE region and the flow region is referred to as the yield point. The co-hydrogels showed physically cross-linked gel structures before the yield points, but showed fluidic structures after the yield points. The yield points were approximately shown as the intersections of the stress–strain curves with the straight line in each of the Figures. Both the shear strain and shear stress at the yield points decreased with the increase of the R_f_-PEG-g-PAA portion from ~10% shear strain for the 100% R_f_-PEG-R_f_ samples (composition #1 in Table 1) to ~4% shear strain for the 12.5% R_f_-PEG-g-PAA/87.5% R_f_-PEG-R_f_ samples (composition #5 in Table 1). In the LE regions, shear strain deformations of the co-hydrogels were small enough so that the gel networks were conserved. However, in the flow region, the larger shear strains dragged the R_f_-cores apart, which subsequently destroyed the hydrogel networks resulting in fluidic solutions of the polymers. After shear strain surpassed the yield point, the shear stress no longer followed the LE trend. In fact, most of the shear stresses even decreased with the increase of the shear strain in the flow regions due to the continued breakdown of the remaining hydrogel network. The shear stresses decreased with the increase of the R_f_-PEG-g-PAA portion for all the hydrogels prepared in water and the buffers, which indicates the decreased strengths of the cross-linkages between the micelles due to the reduced number of cross-linkages between the micelles through the R_f_-PEG-R_f_ chains. As the portion of Rf-PEG-g-PAA increases, the R_f_–groups of R_f_-PEG-g-PAA polymers replaced more of the R_f_-groups of R_f_-PEG-R_f_ polymers which tether the micelle cores together. This experimental phenomenon demonstrates that the R_f_-groups of R_f_-PEG-g-PAA incorporated into the R_f_-cores formed by R_f_-PEG-R_f_. Small-angle neutron scattering (SANS) results show that the aggregation numbers of the R_f_–groups in the R_f_-PEG-R_f_ hydrogels were definite for each combination of the R_f_-group’s molecular weights and PEG’s lengths [24]. Our conclusion makes sense providing that the R_f_-core aggregation number kept unchanged with the incorporation of the R_f_-groups of R_f_-PEG-g-PAA. It is also noticed that the shear stresses have the following general order: hydrogels prepared in the PBS buffer (pH = 7.2) > those prepared in the glycine/NaOH buffer (pH = 10.6) > those prepared in water (pH = 4–5). Stronger shear stress shows stronger gel network interconnection. We will return to discuss the pH dependent shear stress together with the pH dependent moduli in the flowing paragraphs.

**Figure 3:**
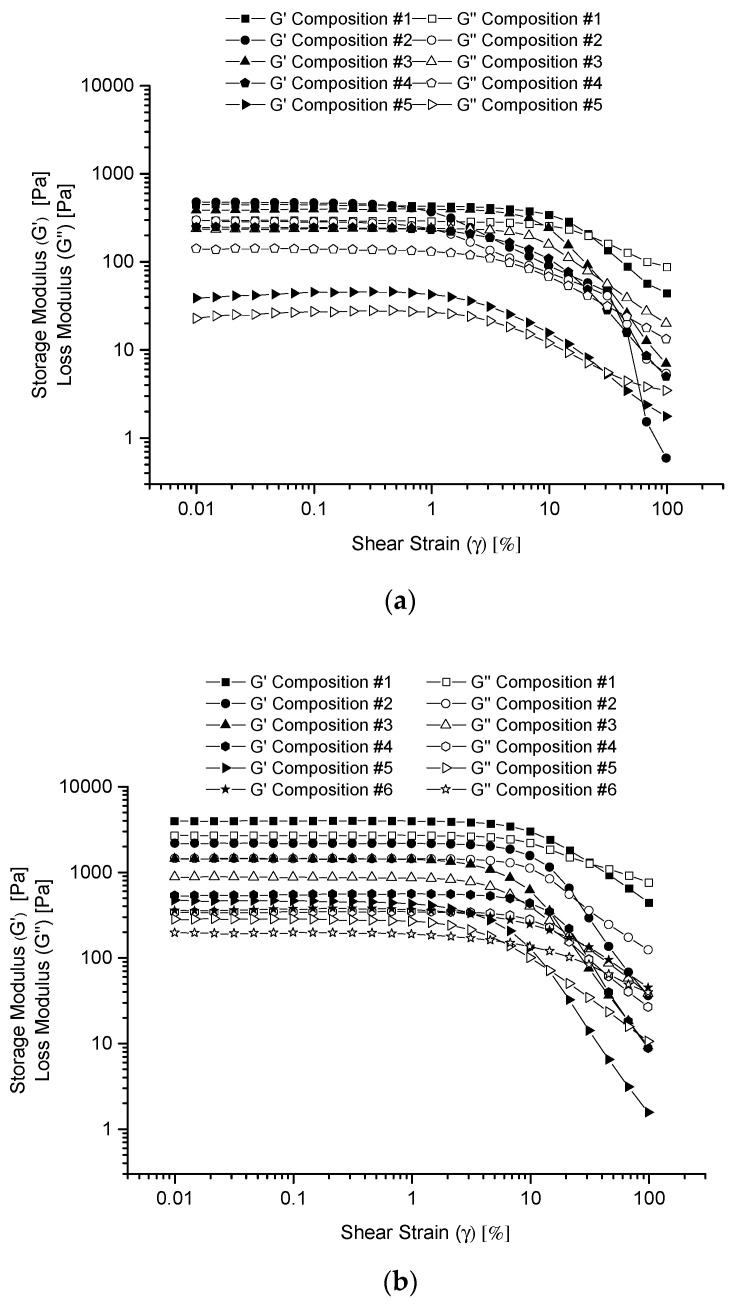

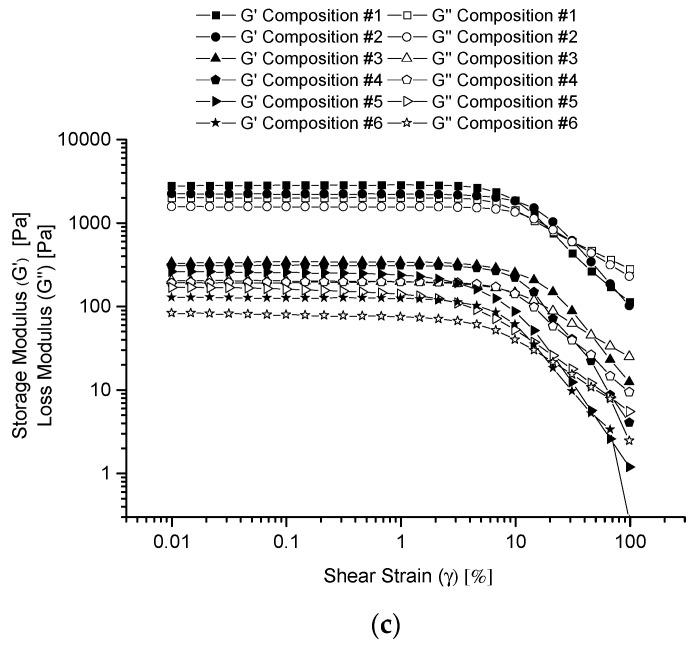
G’ and G’’ versus γ curves (in logarithmic scales) for the given co-hydrogels prepared in water (**a**), PBS buffer (**b**) and glycine/NaOH buffer (**c**).

The above results can be further elaborated by the experimental data of the storage modulus, G’, and the loss modulus, G”, versus the shear strain. G’ describes the component of the elastic property of the co-hydrogel while G” describes that of the viscous property of the co-hydrogel. Figure 3 shows the G’ and G” versus shear strain (γ) curves (in logarithmic scales) for the given co-hydrogels prepared in water (a), PBS buffer (b) and glycine/NaOH buffer (c). The G’ and G” curves show the constant plateau values with small shear strains for all the samples. This kind of plateau region is referred to as the linear viscoelastic (LVE) region. All the G’ values are larger than the G” values for all the samples in the LVE region indicating the gel structures for all the samples. The law of elasticity for oscillatory shear test, $G^{*}=\sqrt{{G^{\prime }}^{2}+{G^{\prime \prime }}^{2}}=\tau /\gamma$, represents the strength of the gel networks. Therefore, the decreased values of G’ and G” with the increased portion of R_f_-PEG-g-PAA in each of the figures indicates the weakened cross-linkages of the co-hydrogel networks, which provide additional proof for the incorporation of R_f_-PEG-g-PAA into the R_f_-PEG-R_f_ hydrogels. With the increase of shear strain for each of the samples, both the G’ and G” curves have gone through the cross-over point (the flow point) where the magnitude of G’ and G” values changed from G’ > G” to G’ < G”. This shows that the co-hydrogel network gradually broke down by the increased shear strain deformation after the flow point.

The influence of pH in the strength of the co-hydrogel networks can also be observed by comparing the curves of the moduli of the co-hydrogels prepared in water and the different pH buffers. The moduli are generally larger for the co-hydrogels prepared in the PBS buffer than those prepared in the glycine/NaOH buffer. The co-hydrogels prepared in water had the smallest moduli. This order is consistent with the observed shear stresses for the co-hydrogels prepared in water and the buffer solutions. At first glance, it seems that because the charges of the PAA block in R_f_-PEG-g-PAA depend on the pH, the pH affects the hydrogen bonding and electrostatic interactions among the PAA and PEG blocks. In water (pH = 4–5), most of the carboxylic acid groups were neutral. In the PBS buffer (pH = 7.2), part of the carboxylic groups were neutral and part of them were negatively charged. In the glycine/NaOH buffer (pH = 10.6), more of the carboxylic acid groups were negatively charged. Therefore, the inter-chain hydrogen bonding and static interactions among the PAA blocks, and between the PAA block and the PEG block would be different at different pH values, which would in turn affect the strength of the gel networks. However, the general trends of the moduli were the same for the 100% R_f_-PEG-R_f_ samples in water and the different buffers where no PAA was involved. Furthermore, the pH values were almost the same for the 100% R_f_-PEG-R_f_ hydrogels prepared in water and in the PBS buffer solution, which indicates that the effect might not be caused by different pH values. Therefore, the ultimate reason can only be the ionic effect of the salts or the conjugate acid-base pairs used to make the buffer solutions. It is known that the R_f_-cores formed by the hydrophobic effect [44]. Thus, the ions in the buffer solutions could make the R_f_-groups associate together more strongly, and it was also possible that more R_f_-groups might form one R_f_-core. 

#### 3.3.2. Frequency Sweep

**Figure 4:**
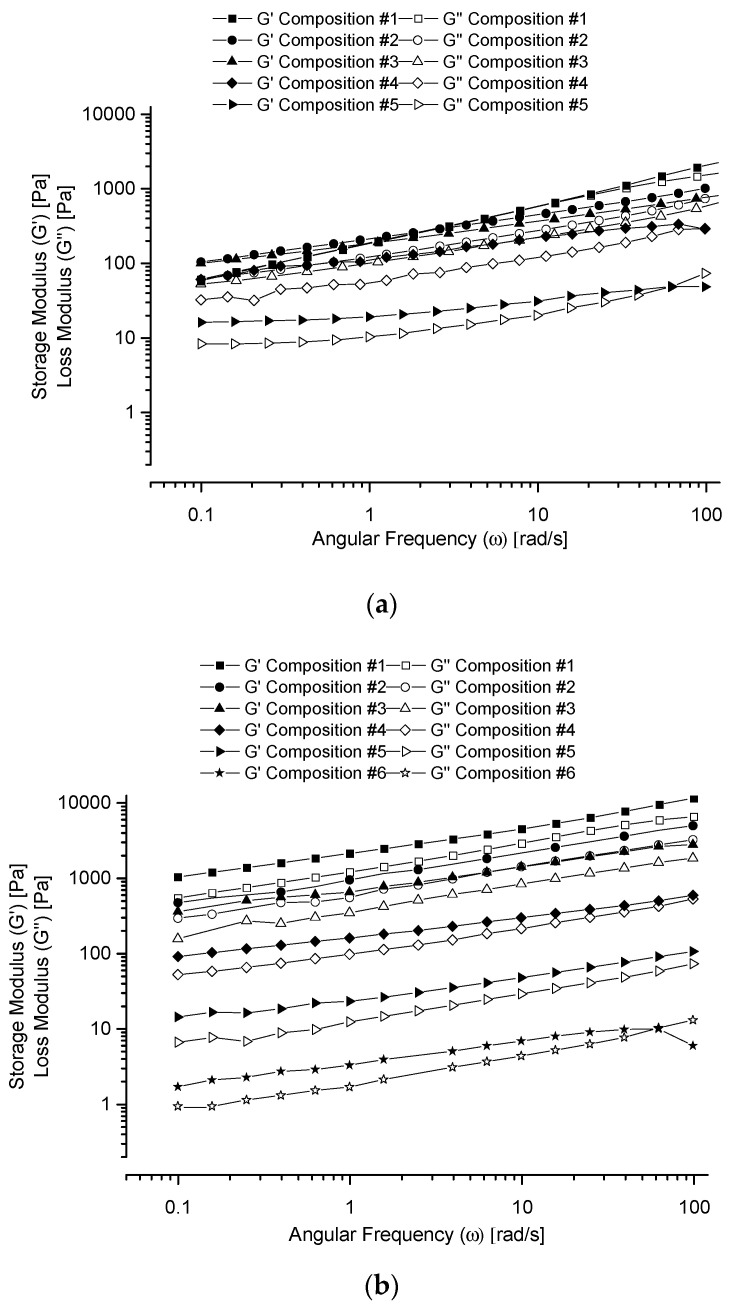

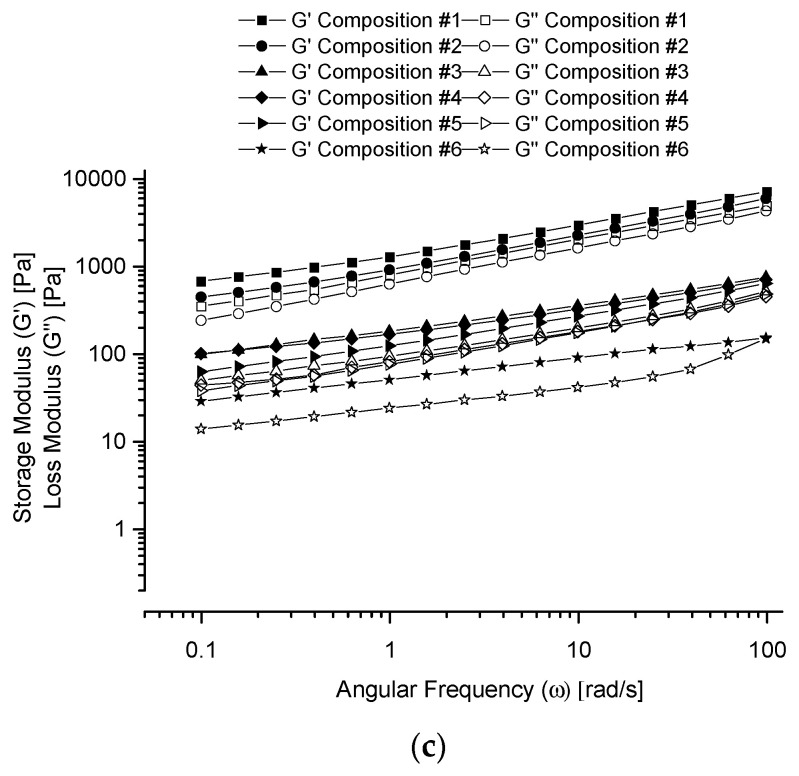
G’ and G’’ versus ω curves (in logarithmic scales) for the given co-hydrogel systems prepared in water (**a**), PBS buffer (**b**) and glycine/NaOH buffer (**c**).

Figure 4 shows the G’ and G’’ versus angular frequency, ω, curves (in logarithmic scales) for the given co-hydrogel systems prepared in (a) water, (b) PBS buffer and (c) glycine/NaOH buffer. All the samples displayed G’ > G’’ curves in the 0.1–100 rad/s angular frequency range with the only exceptions for the samples with the highest R_f_-PEG-g-PAA portions in the ends of the curves. G’ > G” shows the physically cross-linked co-hydrogel networks in the tested angular frequency range. In addition, the moduli increased with the increase of the angular frequency, which demonstrates the physical dispersion stability of the co-hydrogels. Thus, the co-hydrogel materials can stay in 3D shapes with long-term storage stability. The G’ values decreased with the increase of the R_f_-PEG-g-PAA component. G’ shows the cross-linking density. Therefore, with the increase of the R_f_-PEG-g-PAA portion, the number of physical cross-linking between the micelles decreased. However, all of the co-hydrogels could stay in 3D shapes with long-term dispersion stability. This result also indicates that the R_f_-PEG-g-PAA physically incorporated into the R_f_-PEG-R_f_ hydrogel. 

The network of this hydrogel system was self-assembled by individual PEG chains. It is a network at the molecular level, but not one formed by bundles of macromolecules as encountered in many chemically cross-linked hydrogel systems [18]. Therefore, the resolution of the current scanning electron microscopy (SEM) method (in the order of magnitude of μm) may not reveal the molecular-level structures of the co-hydrogel networks. Ideally, fluorescent labeling method [45] would provide direct evidence for the formation of the co-hydrogel system if fluorophores could be used to label the R_f_-groups of R_f_-PEG-R_f_ and R_f_-PEG-PAA. However, our currently used materials and synthetic methods do not allow the straightforward fluorescent labeling to the R_f_-groups. Work in this direction would project a future research of chemistry. This research is to use the tensile test and rheological methods to indirectly address this issue. As discussed above, the experimental results of the tensile test on the control samples, compositions 8 to 10 in Table 1, demonstrate the necessity of the R_f_-group of R_f_-PEG-PAA to associate with the R_f_-cores formed by R_f_-PEG-R_f_. In addition, results of the rheometric measurements demonstrate the viscoelastic properties of the co-hydrogels with different R_f_-PEG-PAA/R_f_-PEG-R_f_ ratios, which in turn verifies the association of the R_f_-group of R_f_-PEG-PAA with the R_f_-cores formed by R_f_-PEG-R_f_.

## 4. Conclusions

R_f_-PEG-g-PAA copolymers were synthesized and integrated into the physically cross-linked R_f_-PEG-R_f_ micellar network through self-assembly. Results of the tensile strength tests demonstrate that the co-hydrogel system possesses pH-sensitive mucoadhesive properties. The mucoadhesion is stronger at low pH (4–5) and high pH (10.6) than in neutral condition, which shows the primary hydrogen bonding interaction and electrostatic interaction between the PAA block and mucin. Experimental results of the control experiments indicate that the R_f_-group of the R_f_-PEG-g-PAA associated with the R_f_-cores of the R_f_-PEG-R_f_ micelles, thus holding the PAA block though the PEG chain on the gel surface. Results of the rheological experiments demonstrate that the R_f_-PEG-g-PAA self-assembled into the R_f_-PEG-R_f_ hydrogel network. The resulting co-hydrogels at different pH conditions could stay in physically cross-linked 3D networks with long-term physical dispersion stability although the number of physical cross-linkages between the micelles decreased with the increase of the R_f_-PEG-g-PAA component. Higher ionic strength in the buffer solutions strengthened the physical association of the R_f_-cores through the hydrophobic effect, thus firming up the interconnection of the micelles through the co-hydrogel network. Results of our co-hydrogel incubation experiments indicate that the co-hydrogels can stay in equilibrium with the sol phases in the water and the buffer solutions. 

Self-assembly of the physically cross-linked hydrogel network of the R_f_-PEG-R_f_/ R_f_-PEG-g-PAA system makes it possible to develop an organic solvent-soluble mucoadhesive hydrogel platform in an aqueous environment with tunable factors including mesh size of the hydrogel network, strength of the physically cross-linked micelles, and binding force of the mucoadhesion through modifying the length of the PEG chain, size of the R_f_ group, length of PAA polymer, and the repeat unit, n, of (R_f_-PEG)_n_- multi-block copolymers for mucosal membrane-localized drug-delivery applications. 

## Figures and Tables

**Table 1:** Compositions of R_f_-PEG-g-PAA, R_f_-PEG-R_f_ (fluoroalkyl double-ended poly(ethylene glycol)), and PAA (poly(acrylic acid)) in the lyophilized solid mixtures to make the co-hydrogels.

Composition #	R_f_-PEG-R_f_ wt%	R_f_-PEG-g-PAA wt%	PAA wt%
1	100.0	0.0	0.0
2	95.0	5.0	0.0
3	92.5	7.5	0.0
4	90.0	10.0	0.0
5	87.5	12.5	0.0
6	85.0	15.0	0.0
7	80.0	20.0	0.0
* 8	95.0	0.0	5.0
* 9	90.0	0.0	10.0
* 10	0.0	5.0	0.0
* 11	0.0	10.0	0.0

* Note: In composition #8, the 5.0% PAA is corresponding to the mass amount of PAA block in composition #2. The actual percentage is 0.9% PAA and 99.1% R_f_-PEG-R_f_. Similarly, in composition #9, the 10.0% PAA is corresponding to the amount of PAA block in composition #4. The actual percentage is 1.9% PAA and 98.1% R_f_-PEG-R_f_. In composition #10, 5.0% R_f_-PEG-g-PAA is corresponding to the amount of R_f_-PEG-g-PAA in composition #2 but without R_f_-PEG-R_f_. In composition #11, 10.0% R_f_-PEG-g-PAA is corresponding to the amount of R_f_-PEG-g-PAA in composition #4 but without R_f_-PEG-R_f_.

## Data Availability

Data is contained within the article and supplementary material. The data presented in this study are available in [Mucoadhesive and Rheological Studies on the Co-Hydrogel Systems of Poly(Ethylene Glycol) Copolymers with Fluoroalkyl and Poly(Acrylic Acid), and the corresponding Supplementary Materials].

## Supplementary Materials

The following are available online at https://www.mdpi.com/article/10.3390/polym13121956/s1: Scheme S1. Route of synthesis of R_f_-PEG-R_f_. Scheme S2. Route of synthesis of R_f_-PEG-g-PAA. Figure S1. Photo picture of the Texture Analyzer experimental setting. Figure S2. The lower plate shows the sample setting of the pig small intestine on the surface of the platform of the texture analyzer. The upper component shows the TA-10 probe on the surface of which a filter paper soaked with R_f_-PEG-R_f_/R_f_-PEG-g-PAA co-hydrogel was glued on. Figure S3. Matrix-assisted laser desorption/ionization-time of flight (MALDI-TOF) mass spectrometry (MS) spectrum of the PEG showing an average molecular weight of 6.2 × 10^3^. Figure S4. MALDI TOF mass spectrum of the R_f_-PEG-R_f_ showing an average molecular weight of 7.4 × 10^3^. Figure S5 MALDI TOF mass spectrum of the R_f_-PEG-OH showing an average molecular weight of 6.8 × 10^3^. Figure S6. MALDI TOF mass spectrum of the R_f_-PEG-g-PAA showing an average molecular weight of 8.0 × 10^3^. Figure S7. ^1^H NMR (nuclear magnetic resonance) spectrum of the R_f_-PEG-g-PAA. The sharp peak at 3.5407 ppm is from the PEG block. Figure S8. Comparison of PAA region of the ^1^H NMR spectrum of PAA (top, blue) and R_f-_PEG-g-PAA (bottom, red). Figure S9. Photo pictures of the sol-gel two-phase coexistences of the 5% R_f_-PEG-g-PAA/95% R_f_-PEG-R_f_ (composition # 2), and the 10% R_f_-PEG-g-PAA/90% R_f_-PEG-R_f_ (composition # 4), respectively, prepared in the following solutions at 37 °C after 11 days: (A) and (B) in PBS buffer (pH = 7.2); (C) and (d) in DI water (pH = 4–5); and (E) and (F) in the glycine/sodium hydroxide buffer (pH = 10.6). Figure S10. Force (g) vs. time (s) curves for the 5.0% mucin Type II sample interacting with the R_f_-PEG-R_f_/R_f_-PEG-g-PAA (here the -g- was omitted in the inset) co-hydrogels and the control samples prepared in water. Figure S11. Bar graph representation of the relative MDSs with respect to the MDS of the water sample for the 5.0% mucin Type II sample interacting with the R_f_-PEG-R_f_/R_f_-PEG-g-PAA co-hydrogels and the control samples prepared in water. Figure S12. Force (g) vs. time (s) curves for the 5.0% mucin Type II sample interacting with the R_f_-PEG-R_f_/R_f_-PEG-g-PAA (here the -g- was omitted in the inset) co-hydrogels prepared in the PBS buffer. Figure S13. Bar graph representation of the relative MDSs with respect to the MDS of the PBS buffer sample for the 5.0% mucin Type II sample interacting with the R_f_-PEG-R_f_/R_f_-PEG-g-PAA co-hydrogels prepared in the PBS buffer. Figure S14. Force (g) vs. time (s) curves for the 5.0% mucin Type II sample interacting with the R_f_-PEG-R_f_/R_f_-PEG-g-PAA (here the -g- was omitted in the inset) co-hydrogel prepared in the glycine/NaOH buffer. Figure S15. Bar graph representation of the relative MDSs with respect to the MDS of the PBS buffer sample for the 5.0% mucin Type II sample interacting with the R_f_-PEG-R_f_/R_f_-PEG-g-PAA co-hydrogel prepared in the glycine/NaOH buffer. Figure S16. Force (g) vs. time (s) curves of the interactions of the various R_f_-PEG-R_f_/R_f_-PEG-g-PAA co-hydrogels and the control samples prepared in water with the pig small intestine surface. Figure S17. Bar graph presentation showing the relative MDSs of various R_f_-PEG-R_f_/R_f_-PEG-g-PAA co-hydrogels and the control samples prepared in water with the pig small intestine surface. Figure S18. Force (g) vs. time (s) curves of the interactions of the various R_f_-PEG-R_f_/R_f_-PEG-g-PAA co-hydrogels and the control sample prepared in PBS buffer with the pig small intestine surface. Figure S19. Bar graph presentation showing the relative MDSs of various R_f_-PEG-R_f_/R_f_-PEG-g-PAA co-hydrogels and the control samples prepared in the PBS buffer with the pig small intestine surface. Figure S20. Force vs. time curves of the interactions of various R_f_-PEG-R_f_/R_f_-PEG-g-PAA co-hydrogels and the control sample prepared in the glycine/NaOH buffer with the pig small intestine surface. Figure S21. Bar graph presentation showing the relative MDSs for various R_f_-PEG-R_f_/R_f_-PEG-g-PAA co-hydrogels and the control samples prepared in glycine/NaOH buffer interacting with the pig small intestine surface.
