# Supplementary material for: Mucoadhesive and Rheological Studies on the Co-Hydrogel Systems of Poly(Ethylene Glycol) Copolymers with Fluoroalkyl and Poly(Acrylic Acid)
Source: Polymers (Basel). 2021 Jun 12;13(12):1956. doi: 10.3390/polym13121956 (PMC8231137; doi:10.3390/polym13121956)
Supplement: Supplementary file 1 [file polymers-13-01956-s001.zip › polymers-1205195-supplementary.pdf]

polymers

Supplementary Materials

Mucoadhesive and Rheological Studies on the Co-Hydrogel Systems of Poly(Ethylene Glycol) Copolymers with Fluoroalkyl and Poly(Acrylic Acid)

Yang Sun, Adiel F. Perez, Ivy L. Cardoza, Nina Baluyot-Reyes and Yong Ba *

Department of Chemistry and Biochemistry, California State University, Los Angeles, CA 90032, USA; youngsoon1987@gmail.com (Y.S.); apere330@calstatela.edu (A.F.P.); icardoz@calstatela.edu (I.L.C.); (N.B.-R.)
* Correspondence: yba@calstatela.edu; Tel.: +1-323-343-2360

Citation: Sun, Y.; Perez, A.F.; Cardoza, I.L.; Baluyot-Reyes, N.; Ba, Y. Mucoadhesive and Rheological Studies on the Co-Hydrogel Systems of Poly(Ethylene Glycol) Copolymers with Fluoroalkyl and Poly(Acrylic Acid). *Polymers* **2021**, *13*, 1956. https://doi.org/10.3390/polym13121956

Academic Editor: Elisabetta Ranucci

Received: 16 April 2021
Accepted: 10 June 2021
Published: 12 June 2021

Publisher's Note: MDPI stays neutral with regard to jurisdictional claims in published maps and institutional affiliations.

Copyright: © 2021 by the authors. Licensee MDPI, Basel, Switzerland. This article is an open access article distributed under the terms and conditions of the Creative Commons Attribution (CC BY) license (http://creativecommons.org/licenses/by/4.0/).

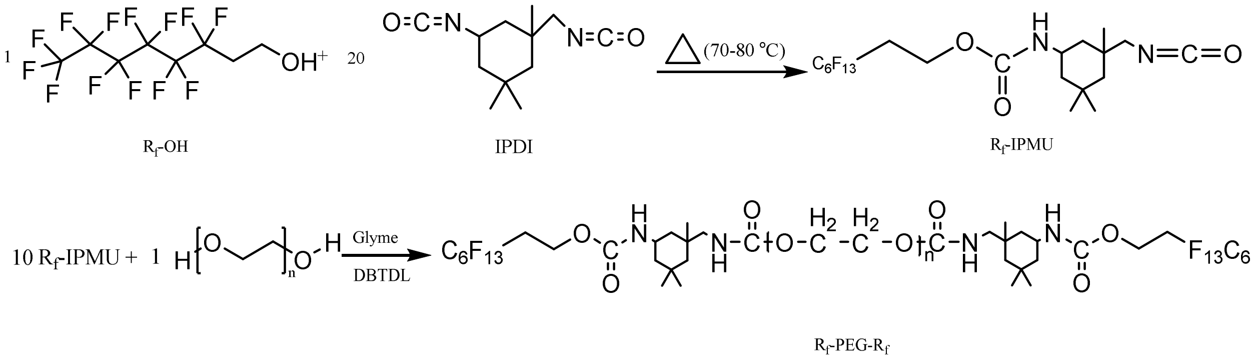

Scheme S1. Route of synthesis of Rf-PEG-Rf.

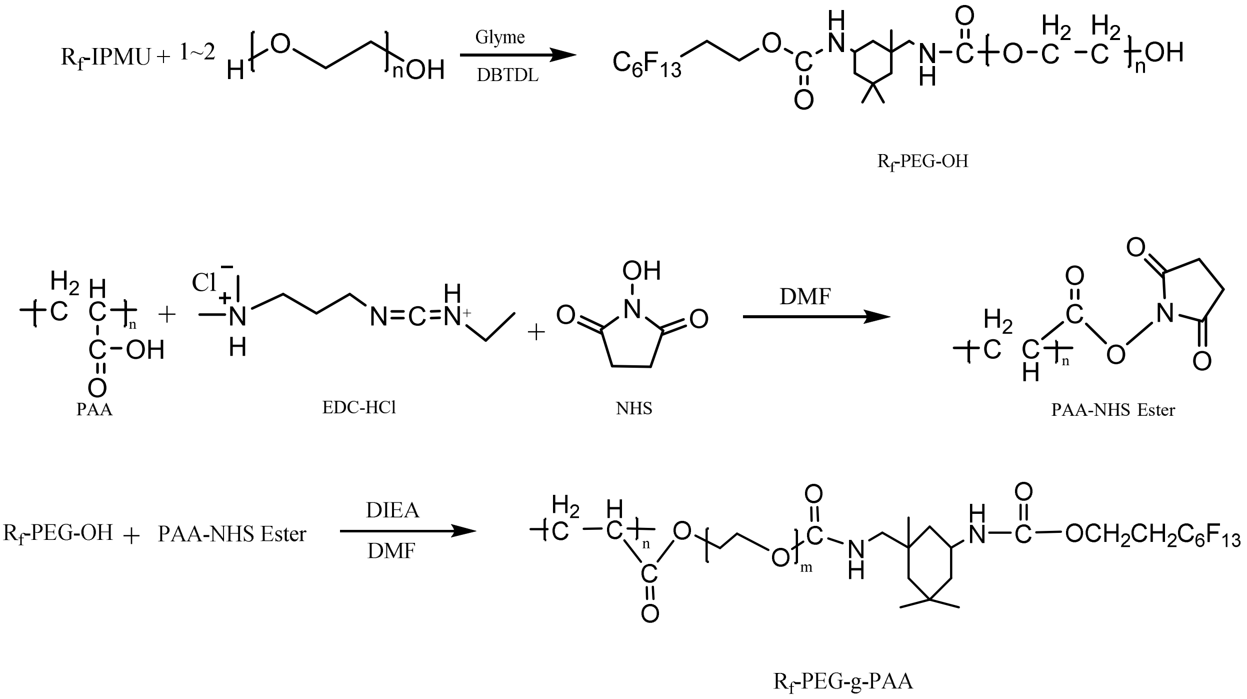

Scheme S2. Route of synthesis of Rf-PEG-g-PAA.

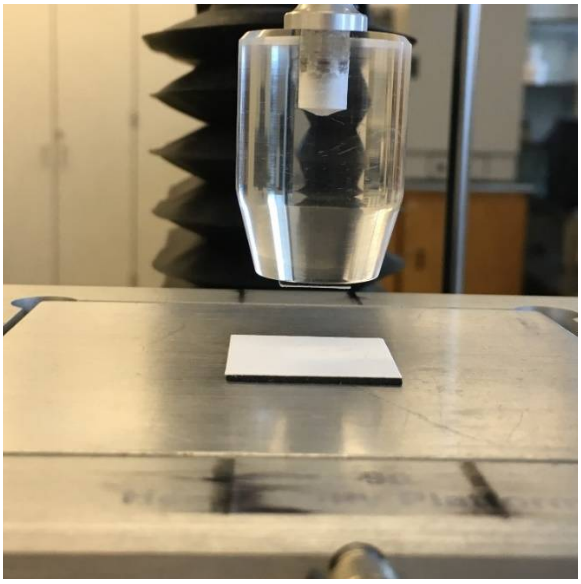

Figure S1. Photo picture of the Texture Analyzer experimental setting.

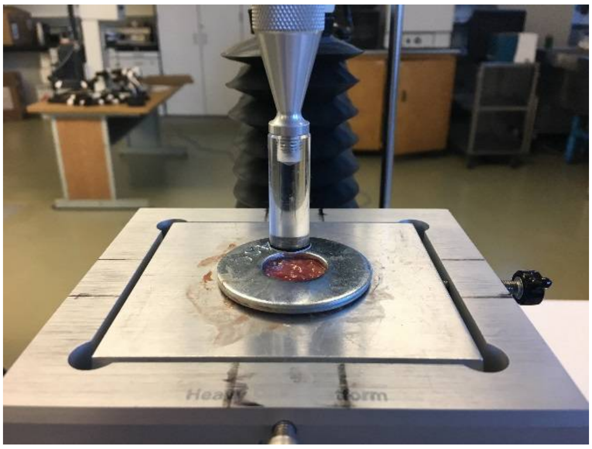

Figure S2. The lower plate shows the sample setting of the pig small intestine on the surface of the platform of the texture analyzer. The upper component shows the TA-10 probe on the surface of which a filter paper soaked with R_f-PEG-R_f/R_f-PEG-g-PAA co-hydrogel was glued on.

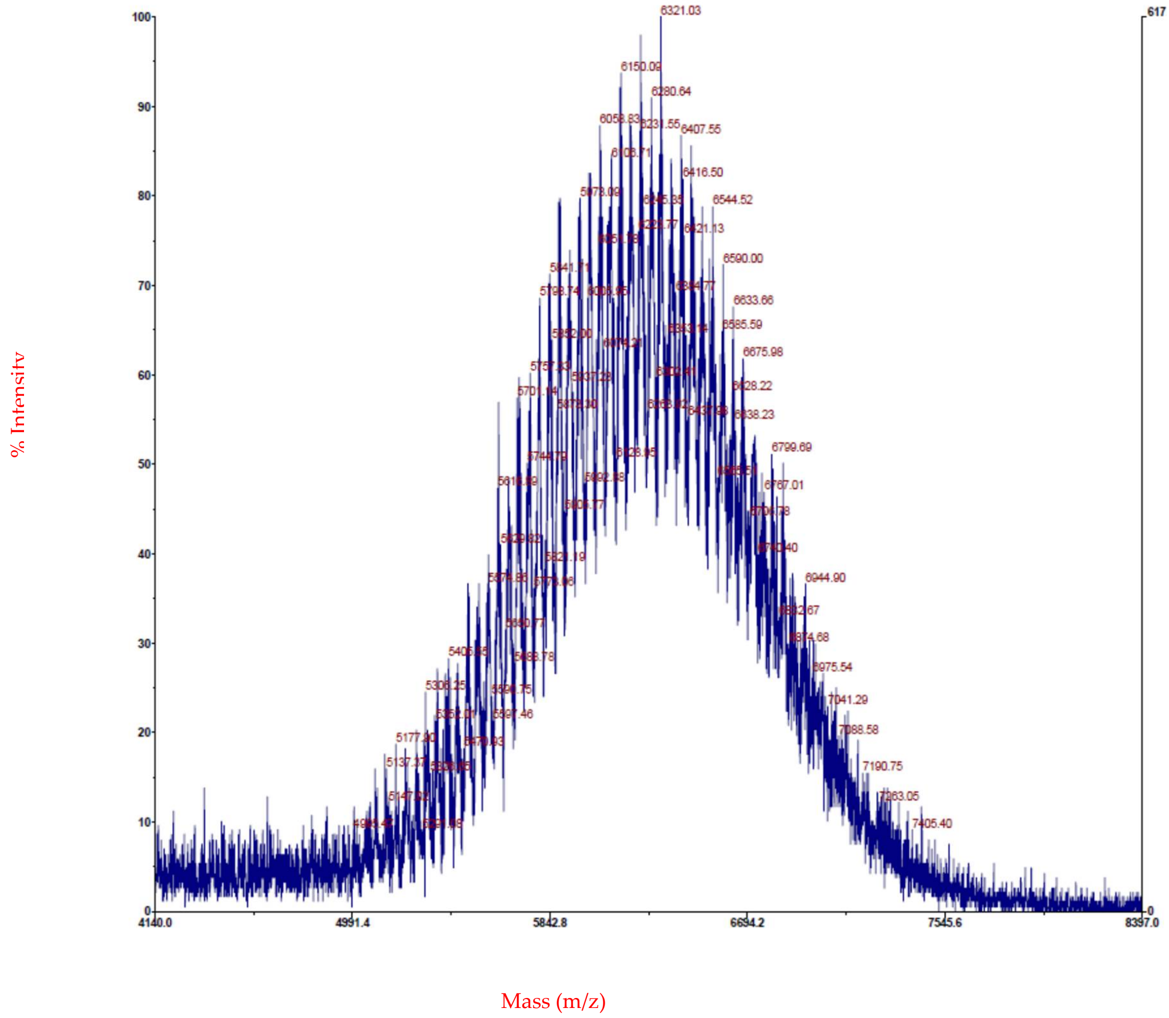

Figure S3. MALDI-TOF MS spectrum of the PEG showing an average molecular weight of $6.2x10^3$.

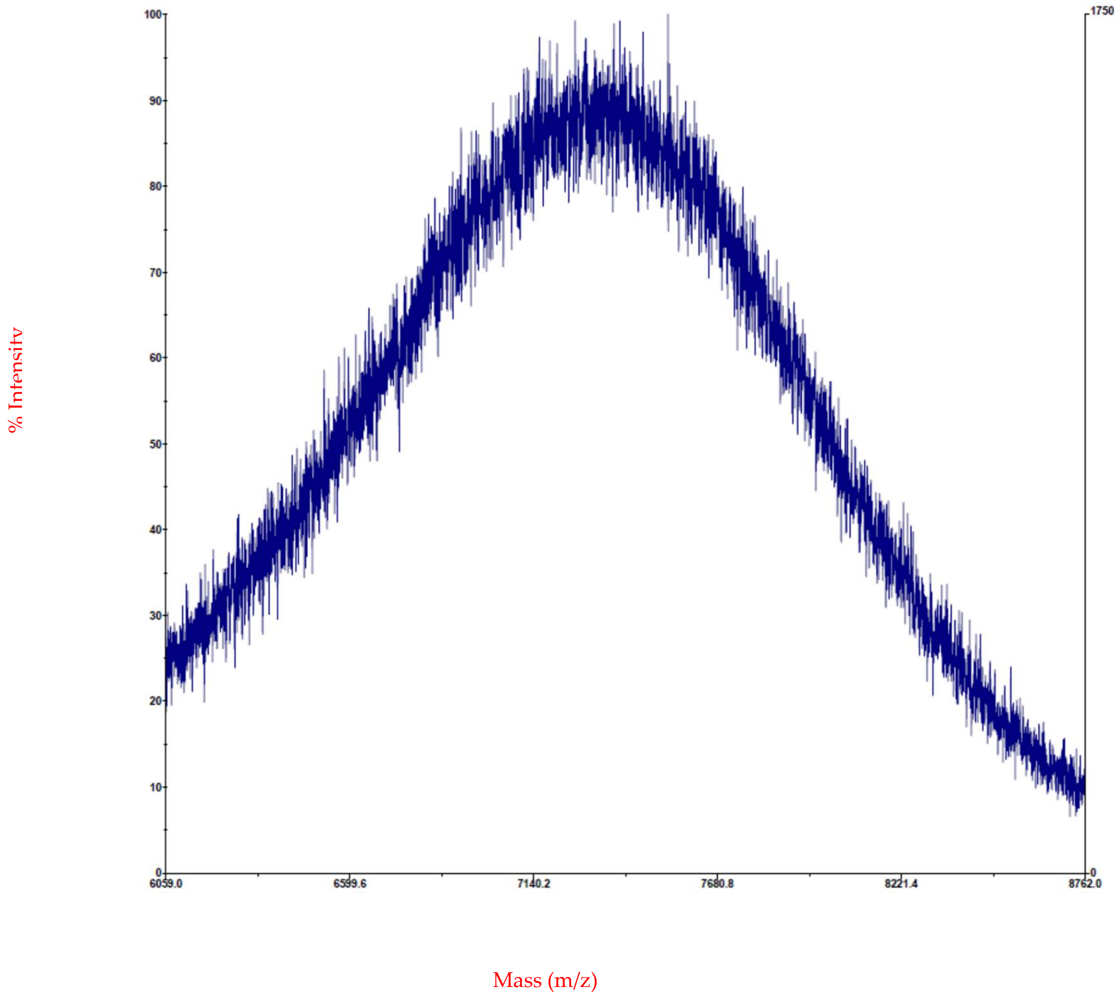

Figure S4. MALDI TOF mass spectrum of the R_f-PEG-R_f showing an average molecular weight of $7.4x10^3$.

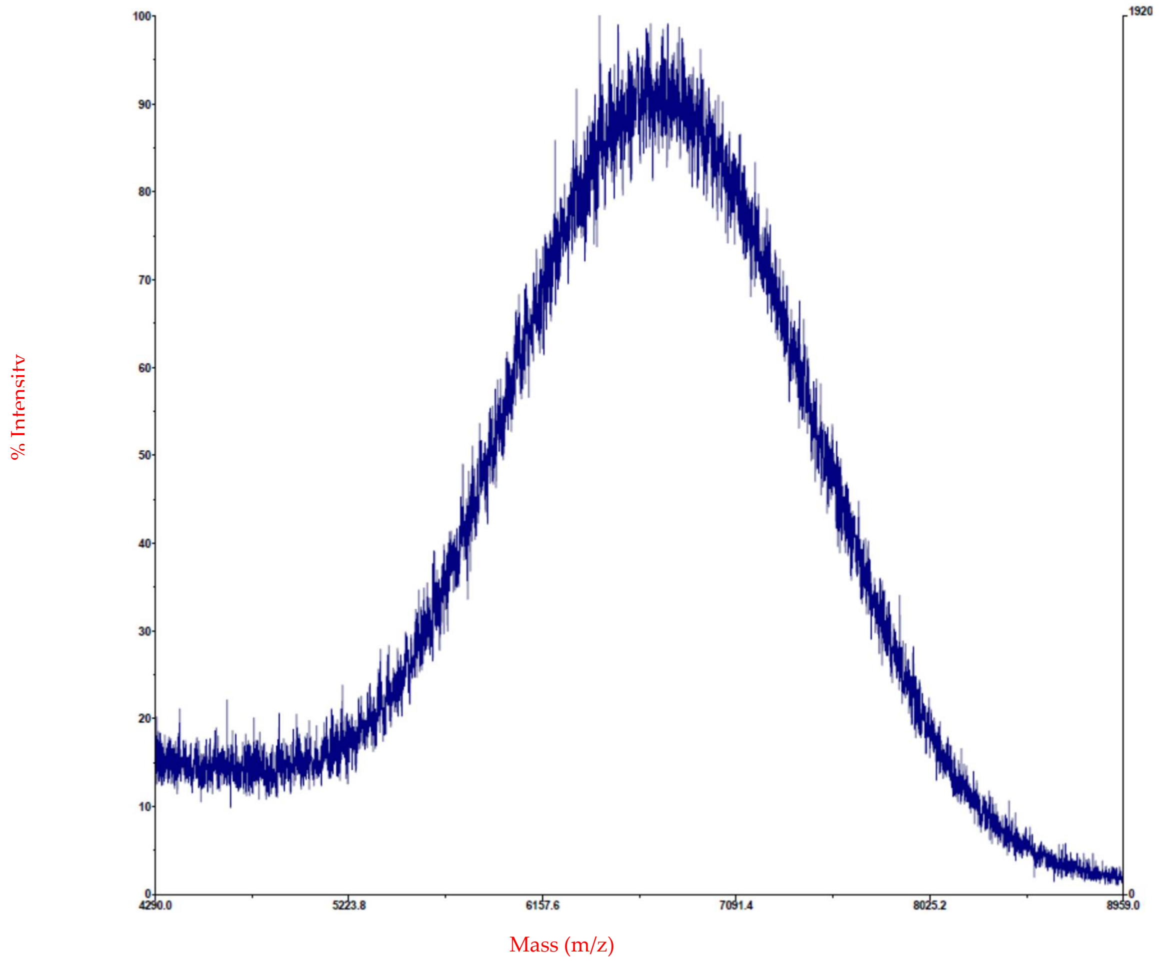

Figure S5 MALDI TOF mass spectrum of the R_f-PEG-OH showing an average molecular weight of $6.8x10^3$.

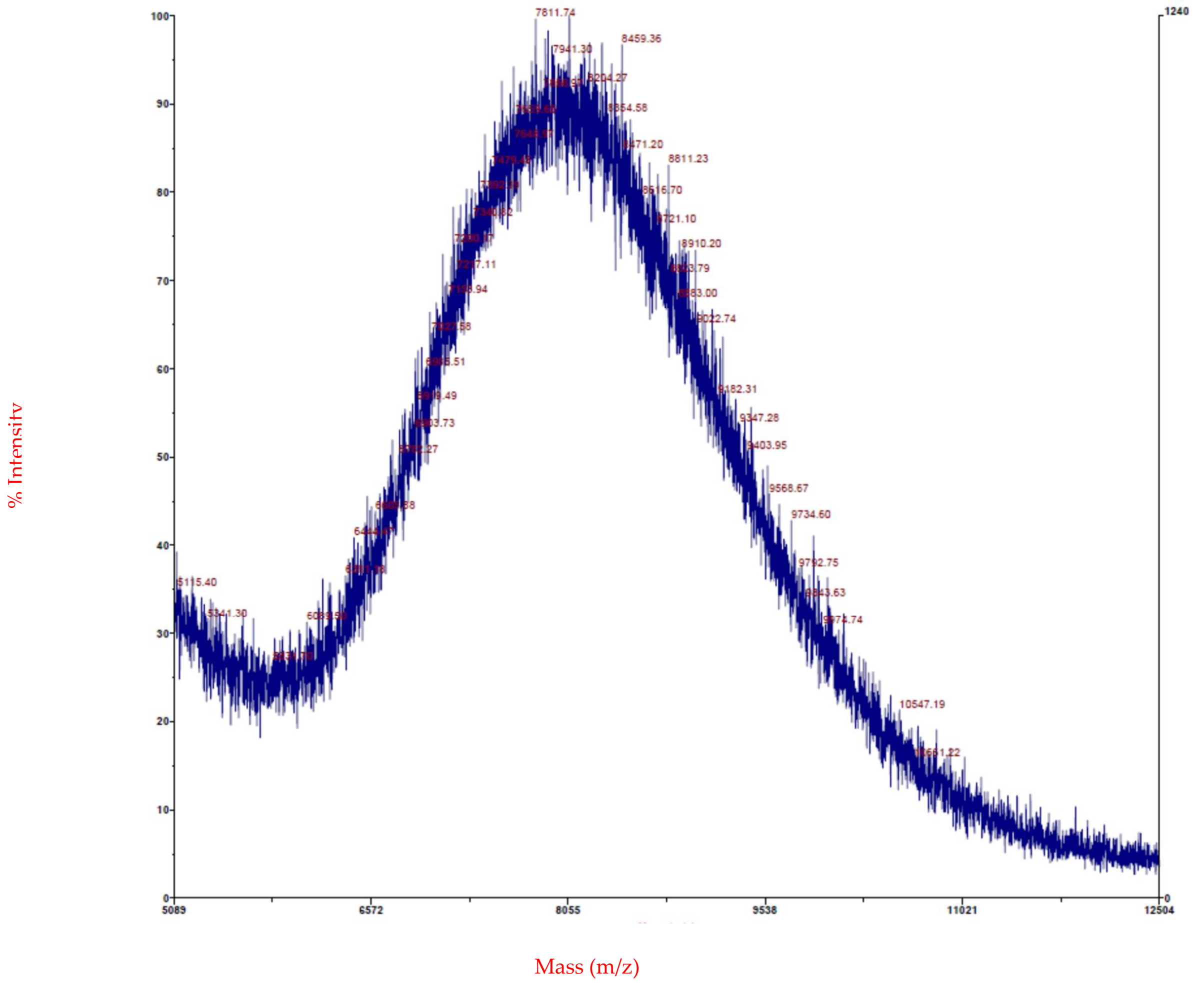

Figure S6. MALDI TOF mass spectrum of the R_f-PEG-g-PAA showing an average molecular weight of $8.0x10^3$.

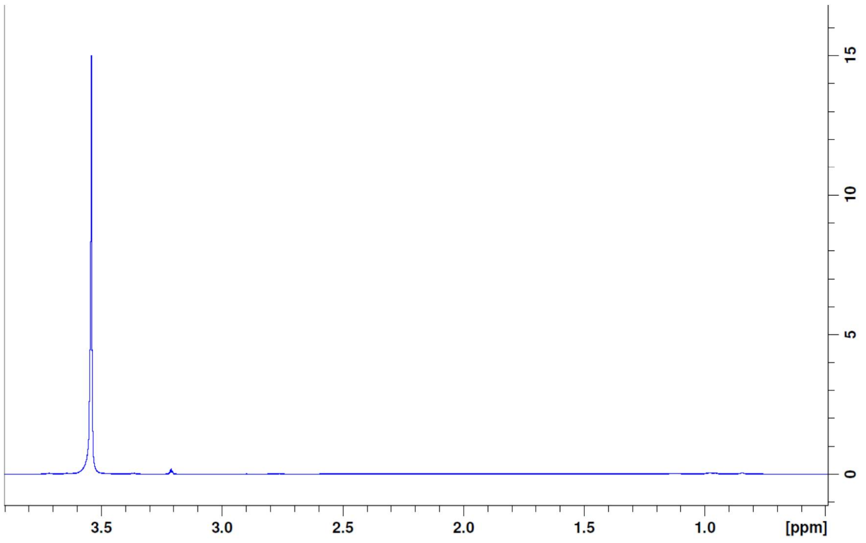

Figure S7. ^1H NMR spectrum of the R_f-PEG-g-PAA. The sharp peak at 3.5407 ppm is from the PEG block.

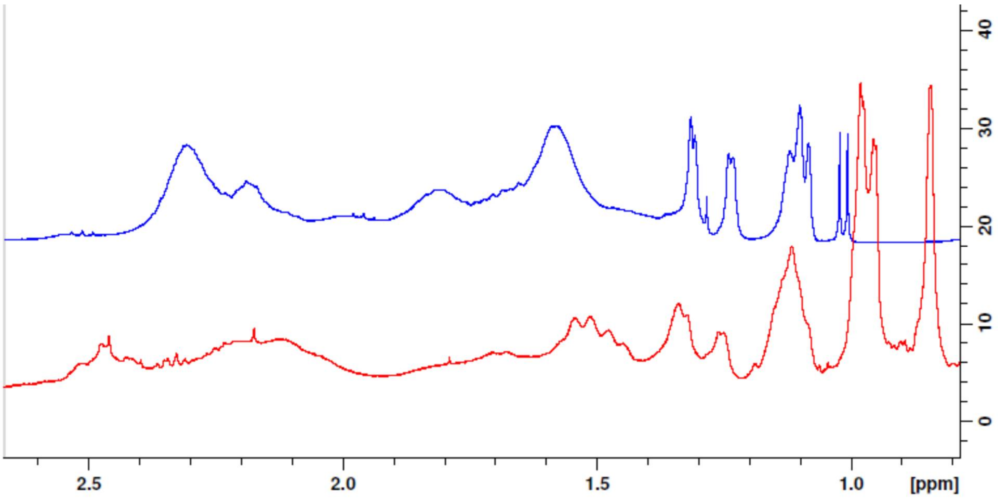

Figure S8. Comparison of PAA region of the ^{1}H NMR spectrum of PAA (top, blue) and R_f-PEG-g-PAA (bottom, red).

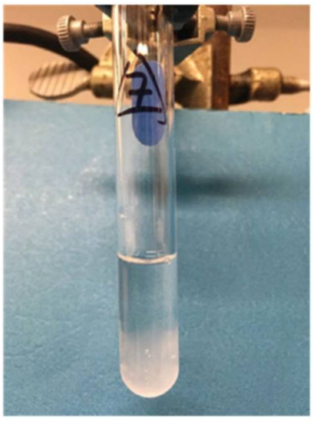

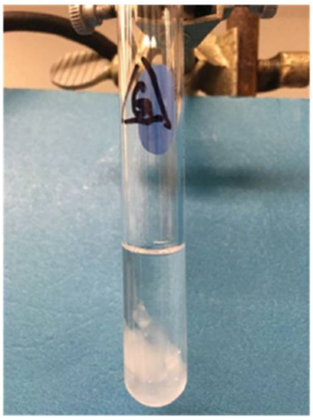

A **B**

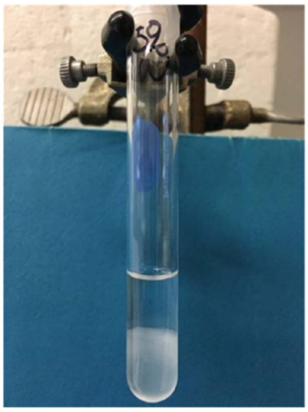

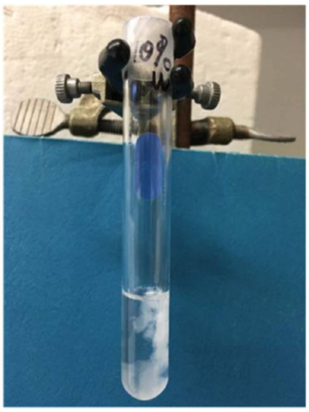

C **D**

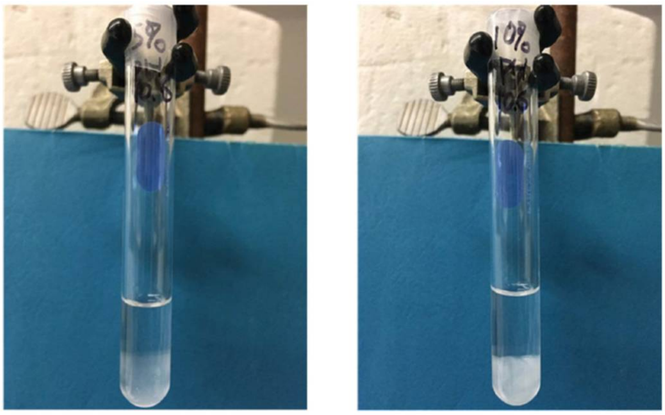

E F

Figure S9. Photo pictures of the sol-gel two-phase coexistences of the 5% R_f-PEG-g-PAA/95% R_f-PEG-R_f (composition #2 in Table 1), and the 10% R_f-PEG-g-PAA/90% R_f-PEG-R_f (composition #4 in Table 1), respectively, prepared in the following solutions at 37 °C after 11 days: (A) and (B) in PBS buffer (pH=7.2); (C) and (d) in DI water (pH=4-5); and (E) and (F) in the glycine/sodium hydroxide buffer (pH=10.6).

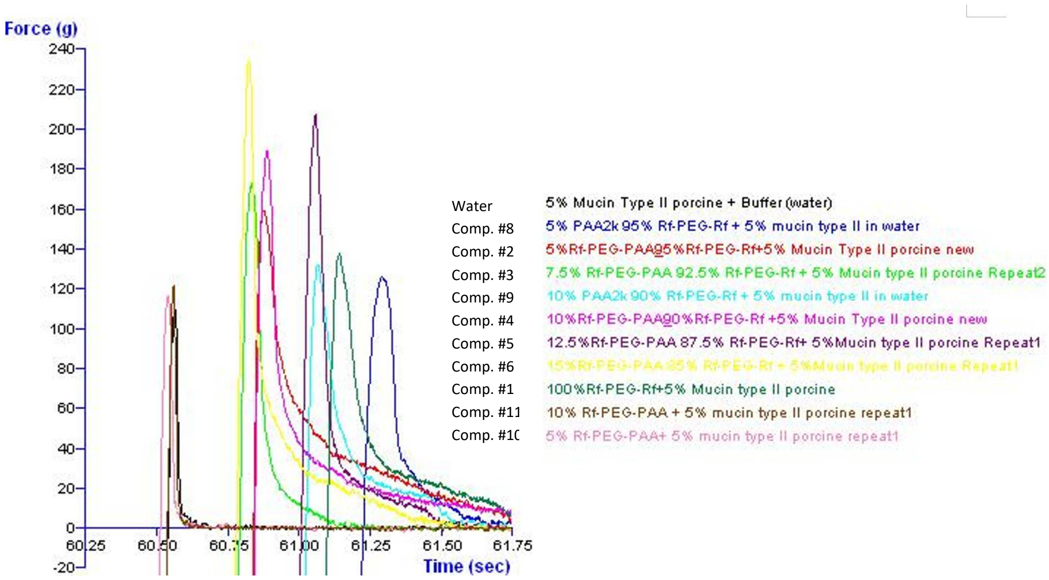

Figure S10. Force (g) vs. time (s) curves for the 5.0% mucin Type II sample interacting with the R_f-PEG-R_f/R_f-PEG-g-PAA (here the -g- was omitted in the inset) co-hydrogels and the control samples prepared in water.

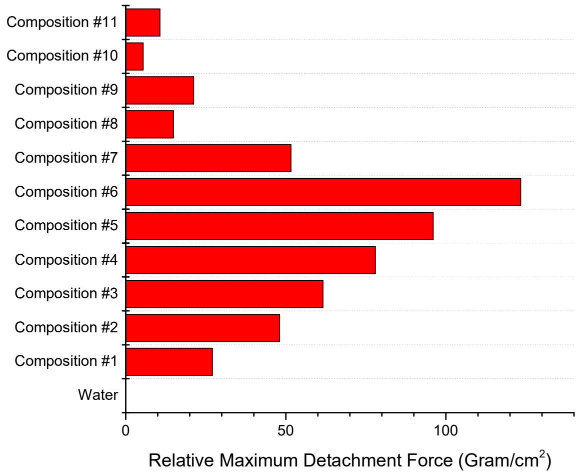

Figure S 11. Bar graph representation of the relative MDSs with respect to the MDS of the water sample for the 5.0% mucin Type II sample interacting with the R_f-PEG-R_f/R_f-PEG-g-PAA co-hydrogels and the control samples prepared in water.

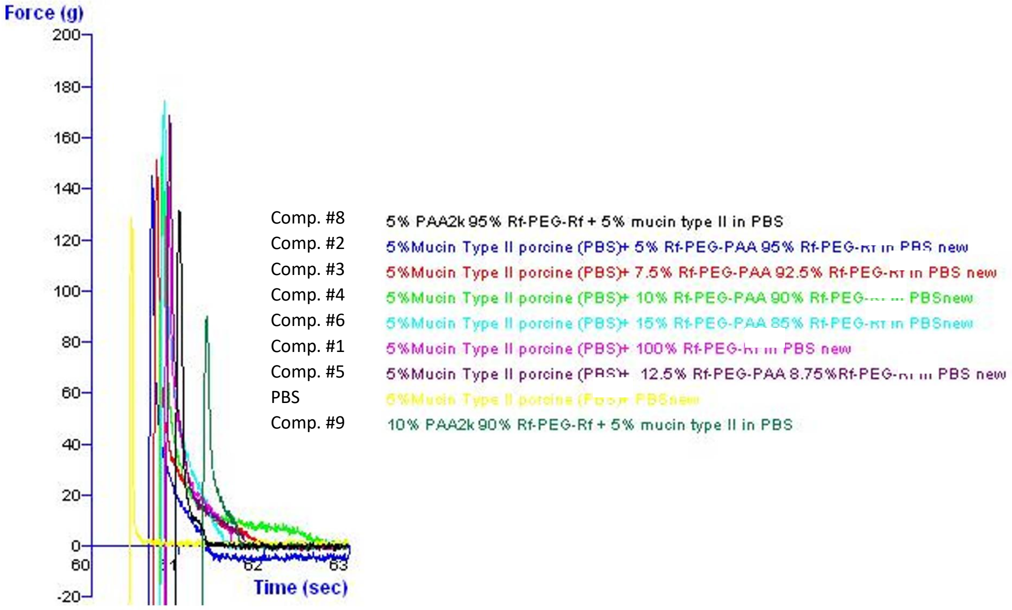

Figure S12. Force (g) vs. time (s) curves for the 5.0% mucin Type II sample interacting with the R_f-PEG-R_f/R_f-PEG-g-PAA (here the -g- was omitted in the inset) co-hydrogels prepared in the PBS buffer.

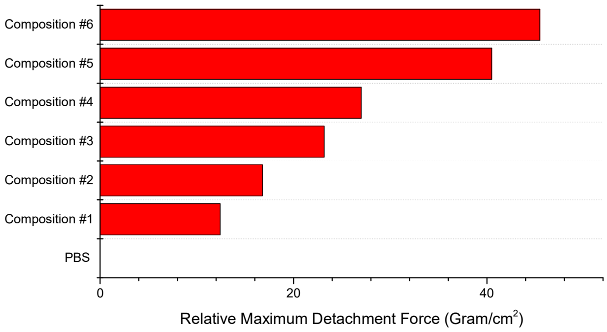

Figure S13. Bar graph representation of the relative MDSs with respect to the MDS of the PBS buffer sample for the 5.0% mucin Type II sample interacting with the R_f-PEG-R_f/R_f-PEG-g-PAA co-hydrogels prepared in the PBS buffer.

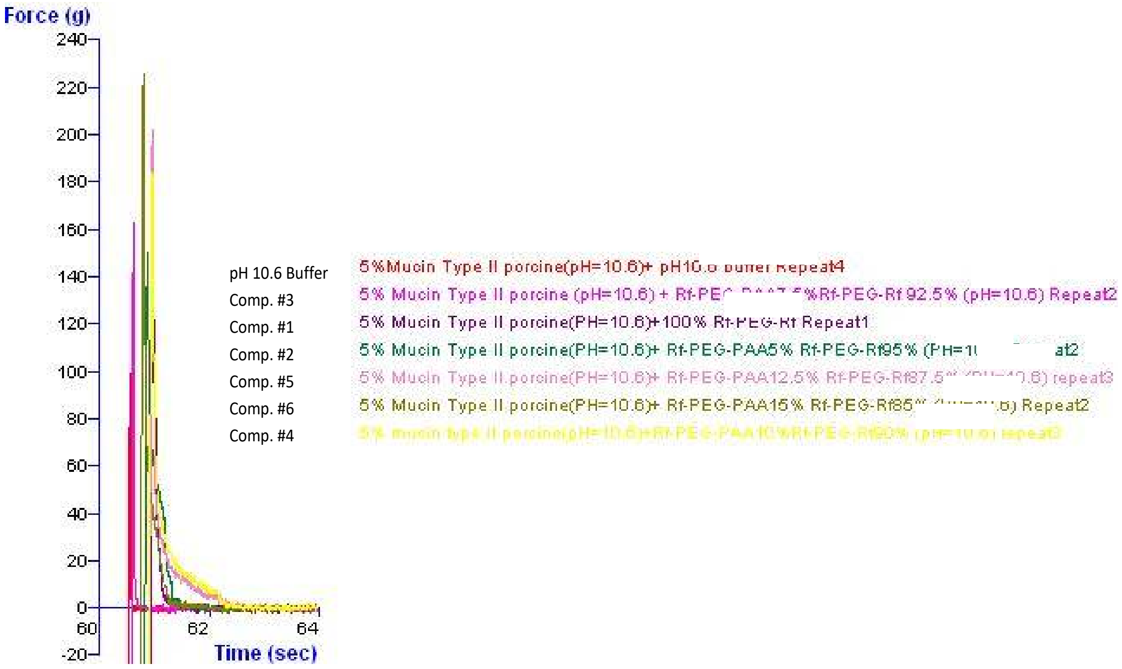

Figure S14. Force (g) vs. time (s) curves for the 5.0% mucin Type II sample interacting with the R_f-PEG-R_f/R_f-PEG-g-PAA (here the -g- was omitted in the inset) co-hydrogel prepared in the glycine/NaOH buffer.

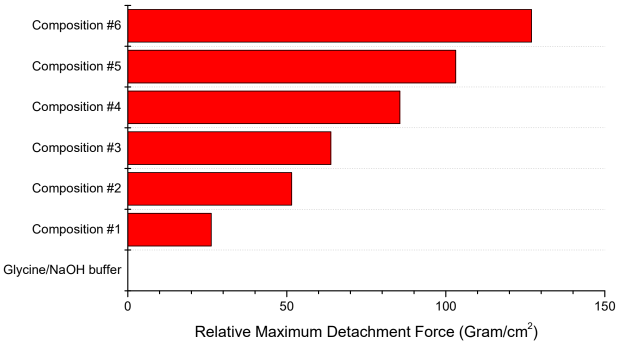

Figure S15. Bar graph representation of the relative MDSs with respect to the MDS of the PBS buffer sample for the 5.0% mucin Type II sample interacting with the R_f-PEG-R_f/R_f-PEG-g-PAA co-hydrogel prepared in the glycine/NaOH buffer.

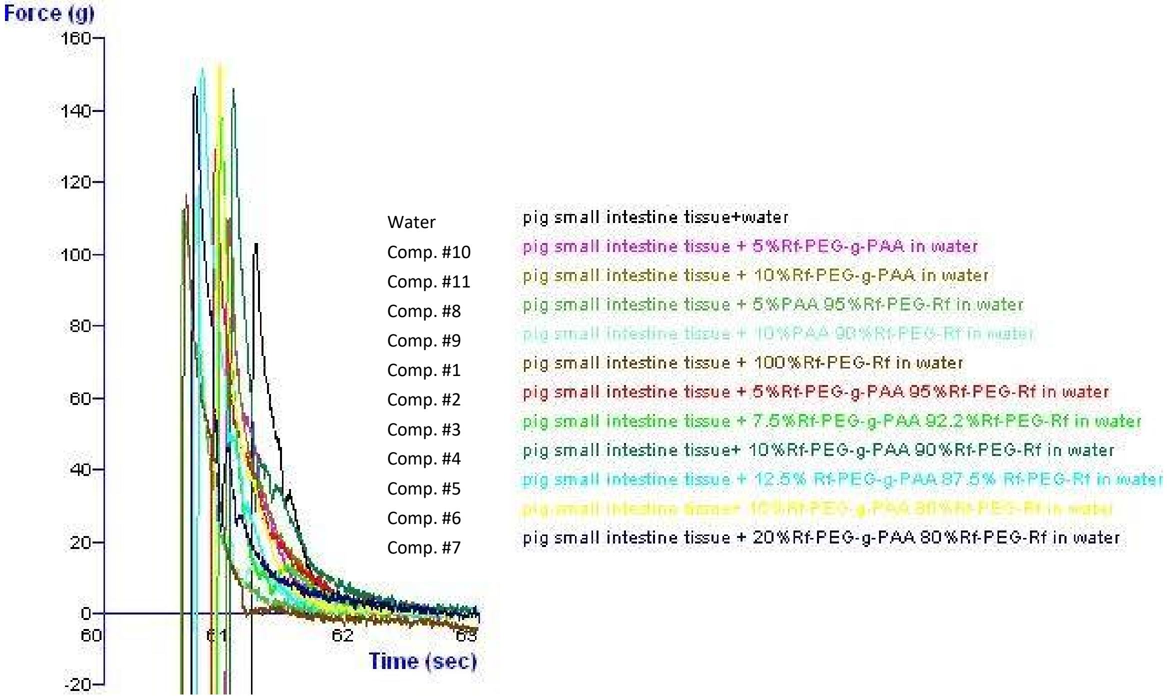

Figure S16. Force (g) vs. time (s) curves of the interactions of the various R_f-PEG-R_f/R_f-PEG-g-PAA co-hydrogels and the control samples prepared in water with the pig small intestine surface.

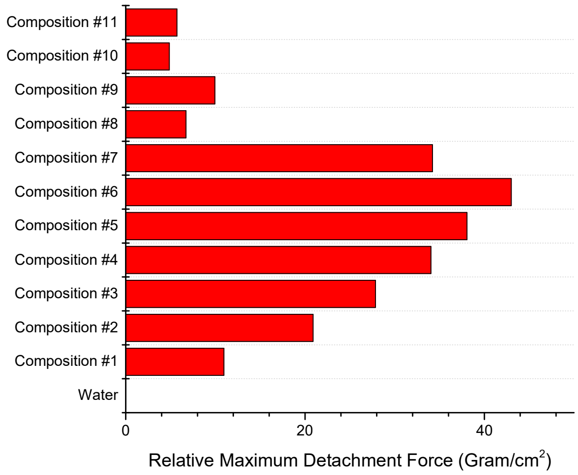

Figure S17. Bar graph presentation showing the relative MDSs of various R_f-PEG-R_f/R_f-PEG-g-PAA co-hydrogels and the control samples prepared in water with the pig small intestine surface.

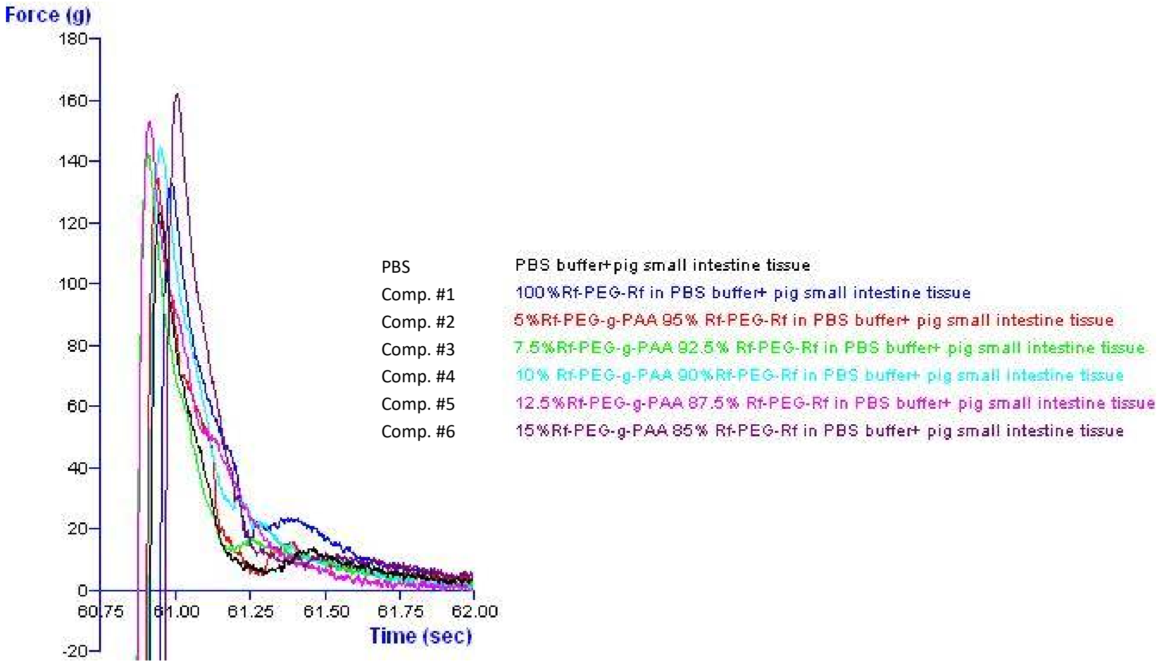

Figure S18. Force (g) vs. time (s) curves of the interactions of the various R_f-PEG-R_f/R_f-PEG-g-PAA co-hydrogels and the control sample prepared in PBS buffer with the pig small intestine surface.

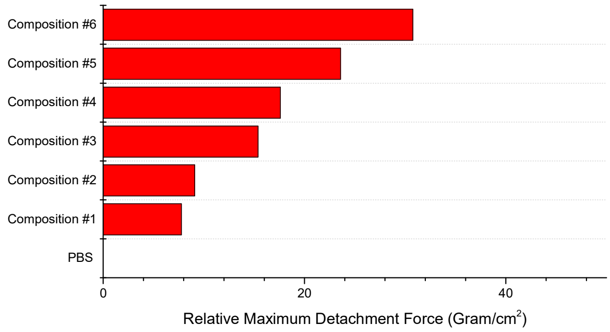

Figure S19. Bar graph presentation showing the relative MDSs of various R_f-PEG-R_f/R_f-PEG-g-PAA co-hydrogels and the control samples prepared in the PBS buffer with the pig small intestinesurface.

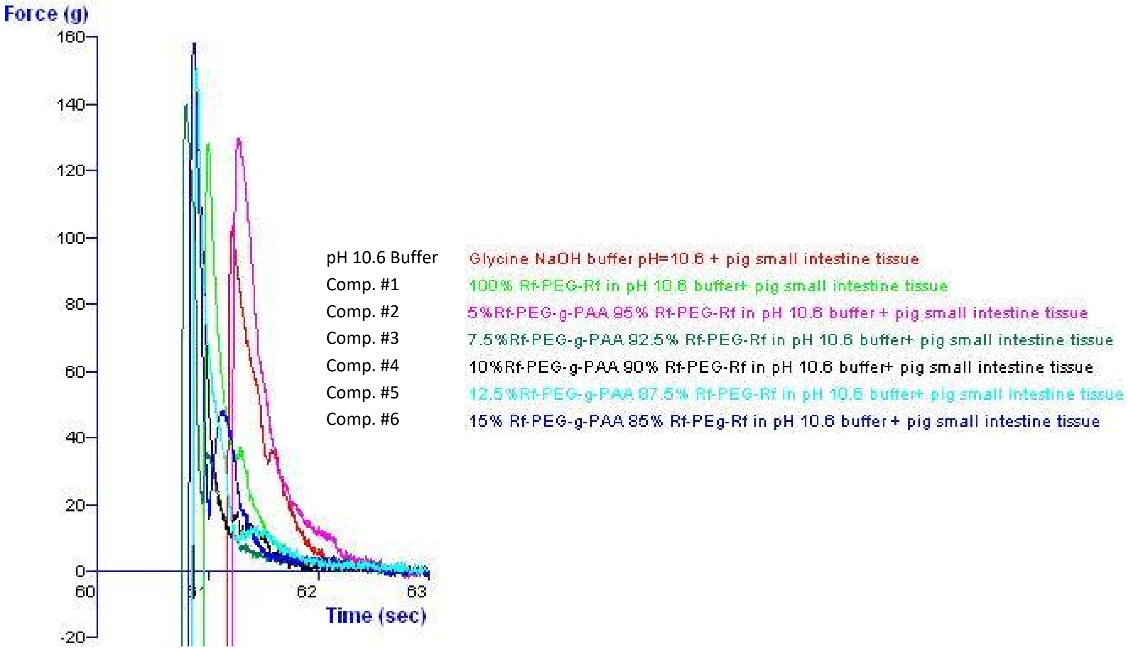

Figure S20. Force vs. time curves of the interactions of various R_f-PEG-R_f/R_f-PEG-g-PAA co-hydrogels and the control sample prepared in the glycine/NaOH buffer with the pig small intestine surface.

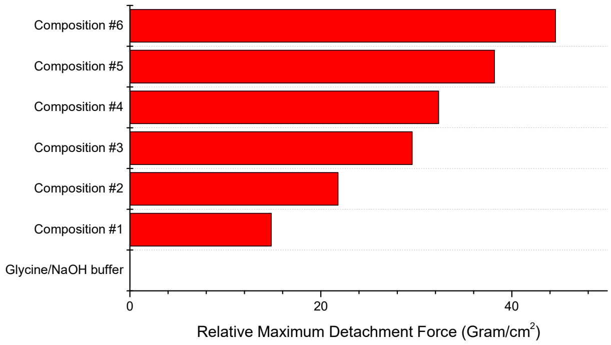

Figure S21. Bar graph presentation showing the relative MDSs for various R_f-PEG-R_f/R_f-PEG-g-PAA co-hydrogels and the control samples prepared in glycine/NaOH buffer interacting with the pig small intestine surface.
